# Whole-genome resequencing reveals genomic footprints of Italian sweet and hot pepper heirlooms giving insight into genes underlying key agronomic and qualitative traits

**DOI:** 10.1186/s12863-022-01039-9

**Published:** 2022-03-25

**Authors:** Salvatore Esposito, Riccardo Aiese Cigliano, Teodoro Cardi, Pasquale Tripodi

**Affiliations:** 1 CREA Research Centre for Cereal and Industrial Crops, S.S. 673, km 25.200, 71122 Foggia, Italy; 2 Sequentia Biotech SL, Carrer d’Àlaba, 61, Barcelona, Spain; 3 CNR-IBBR, Institute of Biosciences and Bioresources, via Università 133, 80055 Portici, Italy; 4 CREA Research Centre for Vegetable and Ornamental Crops, Via dei Cavalleggeri 25, 84098 Pontecagnano Faiano, SA Italy

**Keywords:** *Capsicum annuum*, Resequencing, Italian pepper landraces, Comparative analysis, Repetitive elements, Private variants

## Abstract

**Background:**

Pepper is a major crop species of the Solanaceae family, largely appreciated for its high nutritional and healthy contribution to human diets. In the Mediterranean basin, the favorable pedoclimatic conditions enhanced the selection of several diversified landraces cultivated pepper (*Capsicum annuum*), for whom Italy can be considered a main pole of diversification. Hence, a survey of traditional *C. annuum* genetic resources is essential for deep understanding of such diversity and for applications in genomics assisted breeding. Here, we report whole-genome resequencing analyses of two sweet and two pungent genotypes highly diffused in South Italy and representative of the variability for shape, colour and nutritional properties.

**Results:**

The four genomes were reconstructed at a chromosomal scale using a reference-guided approach, based on a dataset of 2.6 billion paired-end reads, corresponding to 20× genome coverage and a mapping rate above 99% for a final genomes size of approximately 3 Gb. After five iterations of variant calling, a total of 29,258,818 single nucleotide polymorphisms (SNPs) and 1,879,112 InDels, were identified. Substantial differences were observed among the four genomes based on geographical origin, with chromosomes 9 and 11 showing more polymorphisms in the accessions with higher fruit weight and absence of pungency. Among the identified variants, a small private indel (T - > TA) shared between sweet and big fruits accessions induces a frameshift with the generation of a new stop codon in a gene annotated as *extensin*, whereas two private SNPs within hot types were identified in *1-aminocyclopropane-1-carboxylate oxidase* (*ACO*), a key gene involved in fruit ripening. The estimation of repetitive elements highlights a preponderant presence of Long Terminal Repeats (LTRs), the majority of which belonged to *Gypsy* superfamily. By comparing the four genomes with publicly available references including ‘CM334’ and Zunla-1 highlight the presence of 49,475 shared gene families.

**Conclusions:**

The new genomic sequences aim to enrich the whole genome information of pepper local varieties, providing a valuable tool for precision gene mapping, marker discovery, comparative studies. Such knowledge widens the frontiers to understand the selection history of Italian pepper landraces toward the recognition of specificity local agri-food products marks.

**Supplementary Information:**

The online version contains supplementary material available at 10.1186/s12863-022-01039-9.

## Background

Pepper (*Capsicum* spp.) is one of the most important vegetable crops widely recognized for the range of diversity in morphological, agronomical, and quality-related traits and for the presence of its unique pungency of berries due to the presence of capsaicinoids [1]. With 40 million tonnes on a global surface over four million hectares, this crop is among the first thirty food commodities in terms of production (FAOSTAT 2019, 31 June 2021, date last accessed). Pepper belongs to the large Solanaceae family for which several efforts in genome sequencing and resequencing have been done since the second decade of the current century (https://www.solgenomics.net/organism/sol100/view) [2]. The genus includes about 40 species of which five (*C. annuum*, *C. frutescens*, *C. chinense*, *C. baccatum* and *C. pubescens*) have been extensively domesticated in different Latin America regions [3]. Among these, *C. annuum* is the most spread along tropics and temperate zones, where has been subjected to continuous selection leading to the development of several cultivars adapted to diverse environments and appreciated upon consumer preferences.

In the past seven years, genome sequences of domesticated and wild *Capsicum* species have been released. The first Illumina-based whole-genome sequencing and assembly involved the spicy Mexican landrace *C. annuum* cv. Criollo de Morelos 334 (known as CM334) and *C. chinense* PI159236, and both widely used as founders of mapping populations given their resistance to various diseases and pests [4]. In the same year, the genome sequences of the hot pepper *C. annuum* Zunla-1 and the wild progenitor Chiltepin (*C. annuum var. glabriusculum*) were obtained [5]. All studies reported an average genome size of 3.14-3.48 Gb, with about 35 thousand genes and a high percentage of transposable elements (about 80%), the largest of which represented by LTR (Long Terminal Repeat) retrotransposons of the *Gypsy* clade. An improved version of the two reference genomes CM334 and PI159236 along with whole genome assembly of *C. baccatum* PBC81 have been later released estimating the genome size of the latter at 3.9 Gb [6]. By confirming the high proportion of TE elements, the new version highlighted an abundant presence of *Athila* LTRs in PBC81 contributing species-specific genome expansion in the Baccatum lineage as well as several chromosomal rearrangements that differentiated *C. baccatum* from the other species, revealing evolutionary relationships and estimated lineage-divergence times in pepper. Furthermore, while the number of genes was confirmed in PI159236, the comparison between the two versions of CM334 revealed differences for protein coding gene annotation within ~ 10,000 genes, most of which falling in TEs in the first genome release. The linked-read sequencing has been applied in *C. annuum* to sequence F_1_ heterozygous individuals from a cross between CM334 and a non-pungent blocky accession [7]. Although genome assembly was comparable with the available genomes, the Chromium 10x technology allowed to generate a highly ordered and more contiguous sequence assembly with respect to the available *C. annuum* reference genomes, better resolving marker positioning in pericentromeric regions. Anyhow, this genome still lack annotation making it not suitable for resequencing studies. More recently, two additional genome assemblies and annotations of *C. annuum* have been generated in order to unveil the evolutionary mechanism underlying the variation of nucleotide-binding and leucine-rich repeat genes [8].

Despite the progress achieved in de novo genome assemblies, minor efforts have been done so far in re-sequencing, making such knowledge limited respect to other crops for which hundreds of accessions have been already re-sequenced: i.e., tomato [9], bean [10], rice [11], soybean [12]. While a first attempt to resequencing wild and cultivated pepper accessions have been done by Qin et al. [5] to provide new insights about genes involved in pepper domestication, further efforts involve the resequencing to discover new genomic loci as well as investigate functional variation within genes conferring resistance against main biotic stress including bacterial wilt [13] and powdery mildew [14]. Local varieties have been instead re-sequenced by Acquadro et al. [15] for determining structural and functional variation within the genomes of typical landraces from northern Italy.

This study reports the resequencing of four widely recognized Italian landraces representative of the variation for fruit shape (spherical, horn and blocky), colour (red and yellow), nutritional properties (e.g., vitamins, pungency level). Transcripts were assembled with a reference-based approach prior to genome annotation. We estimated the repetitive elements and functionally classified variants putatively associated with the origin of divergence and underlying gene of agronomic and quality interest. We then inferred the presence of private SNPs occurring within each landrace. Based on their distribution, SNPs are considered private when are fixed in a single or a range of samples of a collection and absent in the rest [16] due to mechanisms (e.g., adaptation to environmental pressure, human-based selection) that shape the genetic makeup of cultivars. Despite private SNPs are not reported to be crucial for determining major changes of plant phenome [17], their investigation could highlight the existence of potential candidates for traits of agricultural interest. Finally, comparative analysis with publicly available genomes has been carried out. These reconstructed assemblies based on resequencing contribute to improve the knowledge of whole genome sequences in *C. annuum*.

## Results

### Phenotyping profiles

Plant material consisted of four local varieties with contrasting phenotypic characteristics and diverse geographical origins. Two sweet genotypes were retrieved from the Campania region in South Italy: the horn-shaped type “Corno di Toro” (hereafter CDT) and the roundish type “Papaccella” (hereafter PAP). Two pungent types were retrieved from Calabria region, also in South Italy: the horn-shaped “Sigaretta” (known also as Diavolicchio, hereafter SIG) and the cherry-shaped “Ciliegino” (hereafter CIL). All accessions were red coloured except for “Corno di Toro” with yellow colour at maturity (Fig. S1).

A wide range of phenotypic variation is represented by the selected landraces (Table 1). Sweet accessions (CDT and PAP) are characterized by higher productivity and major fruit weight respect to the hot types (SIG and CIL). Total yield per plant ranges from 360.11 (SIG) g to 2315.53 g (CDT) while fruit weight ranges from 4.62 g (CIL) to 104.20 g (PAP). The variability of fruit shape is confirmed by quantitative measurements and FS index which ranges from 0.64 (PAP) to 6.78 (SIG). CIL, a typical landrace from Calabria region used as fresh, dried or processed (e.g., canned with olive oil), is characterized by cherry-like fruits with length and diameter less than 2.0 cm and 2.5 cm, respectively. The other chilli landrace from Calabria, SIG, is a typical small horn used for fresh and powder consumption with a very low fruit width (< 1.5 cm) and thin pericarp thickness (< 0.5 cm). On the contrary, the two sweet types from Campania region are established cultivars grown since the beginning of the twentieth century and highly appreciated by end-users for the crunchy pulp and a very aromatic and characteristic flavor. PAP have fruits squashed at the ends and costated, being famous in the local cuisine as a characteristic ingredient of many traditional recipes being prepared with tomato sauce or filled and served as a side dish. CDT is a typical horn pepper pointed at the apex with 1-2 lobes and a sweet taste.

**Table 1:** Agronomic, chemical, and biochemical characteristics of the four landraces studied

Trait	Acronym^a^	CDT	PAP	CIL	SIG
Total Yield	TY	2315.53 ± 772.84	1596.48 ± 443.70	456.03 ± 135.30	360.11 ± 115.56
Fruit Weight	FW	76.28 ± 12.14	104.20 ± 13.74	4.62 ± 0.45	4.89 ± 0.82
Fruit Lenght	FL	15.45 ± 1.25	4.72 ± 0.35	1.83 ± 0.12	9.10 ± 1.08
Fruit Width	FD	5.30 ± 0.36	7.34 ± 0.33	2.37 ± 0.22	1.34 ± 0.11
Fruit Shape	FS	2.92 ± 0.22	0.64 ± 0.02	0.78 ± 0.06	6.78 ± 0.62
Pericarp Thickness	PT	3.75 ± 0.37	4.93 ± 0.36	0.69 ± 0.67	0.31 ± 0.27
L^a^	L^a^	52.02 ± 2.21	32.09 ± 1.84	35.76 ± 1.96	34.37 ± 1.60
a^a^	a^a^	14.47 ± 3.26	21.59 ± 0.78	29.71 ± 4.38	32.82 ± 0.89
b^a^	b^a^	49.52 ± 3.08	13.84 ± 0.32	17.26 ± 6.70	21.39 ± 1.32
Chroma	Chroma	51.65 ± 3.43	25.64 ± 0.74	34.56 ± 6.90	39.20 ± 0.25
Soluble Solids	SSC	8.10 ± 0.88	7.55 ± 0.34	8.85 ± 2.08	8.86 ± 2.26
pH	pH	5.37 ± 0.30	5.53 ± 0.14	5.04 ± 0.22	5.19 ± 0.13
TA%	TA%	0.19 ± 0.03	0.16 ± 0.06	0.08 ± 0.04	0.07 ± 0.04
Ascorbic Acid	AsA	106.37 ± 16.91	125.50 ± 20.11	165.73 ± 35.68	127.12 ± 57.31
a-tocopherol	a-toc	7.26 ± 3.50	8.64 ± 2.83	6.31 ± 1.47	10.26 ± 3.19
Capsaicin	Caps	0.00 ± 0.00	0.00 ± 0.00	868.53 ± 206.26	2352.75 ± 603.84

^a^TY is expressed in grams per plant; FW is expressed in grams per fruit; FL, FD and PT are expressed in centimeters; SSC is expressed as °Brix; TA is expressed as mEq % fresh weight; ASA and a-toc, are expressed as mg/100 g fresh weight; Caps is expressed as mg/kg dry weight

**Fig. 1:**
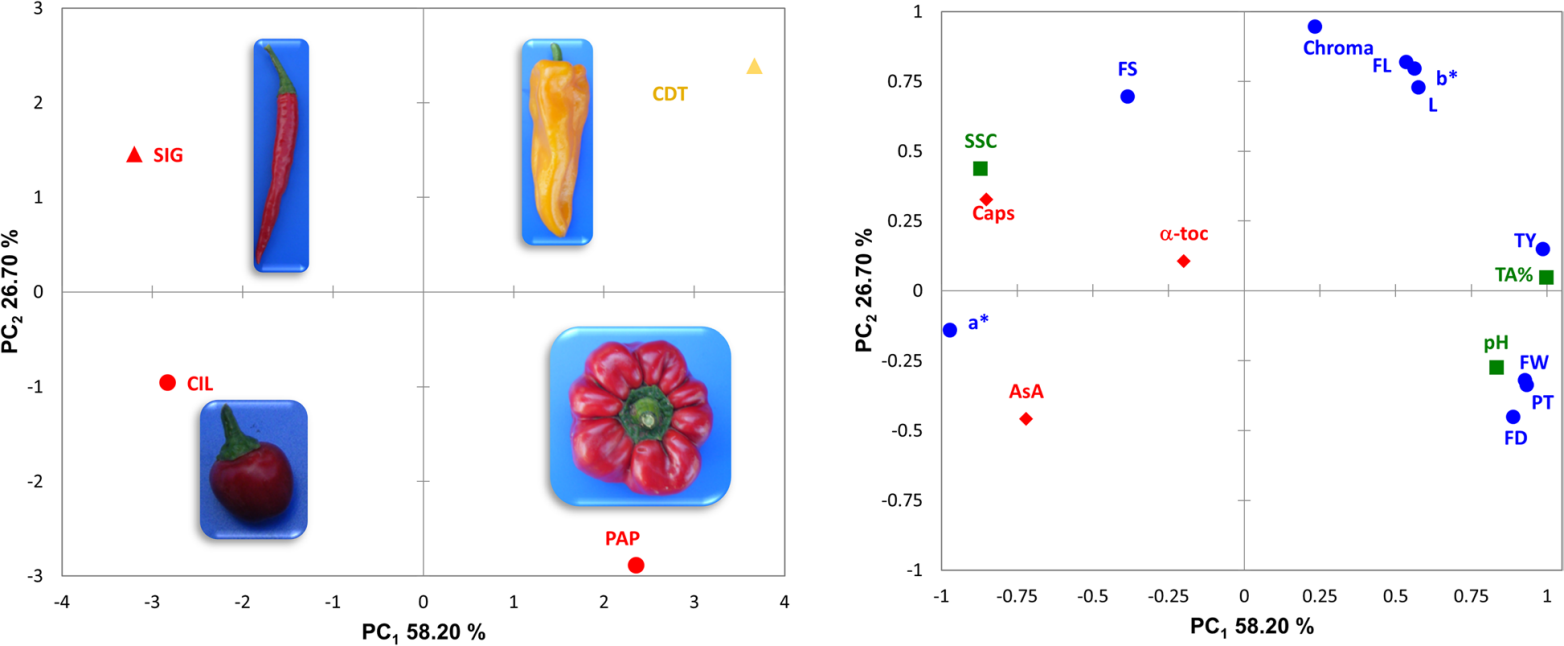
Whole phenotypic variability of the four *C. annuum* genotypes. Loading plot of the first (PC_1_) and second (PC_2_) principal components showing the variation for 16 morpho-agronomic and phytochemical traits scored in the four landraces. Based on fruit shape and colour at maturity accessions are represented by different coloured symbols: circles indicate roundish types, triangles indicate horn types. On the right are indicated the distribution of the traits scored. The direction and distance from the centre of the biplot indicate how each OTU contributes to the first two components. The different category of traits is indicated using different colour codes as following: i) morpho-agronomic and colour traits, blue with dots; ii) chemical traits, green squares; iii) bioactive compounds, red rhombi. For trait acronyms, see Table 1

The two hot types show higher average levels of soluble solids and pH and lower average total acidity than sweet types. Interestingly, vitamin C is higher in the hot types (165.73 mg/100 g FW for CIL, and 127.12 mg/100 g FW for SIG) while vitamin E is more genotype-dependent being lower in CIL (6.31 mg/100 g FW) and higher in SIG (10.26 mg/100 g FW). The projection of the accessions on the biplot of the two first principal components (PCs) highlights how the accessions are independently positioned in either the positive and negative axis of the first and second PCs (Fig. 1). SIG stands out for its high pungency, vitamin E and soluble solids contents, CIL for the content of vitamin C, CDT for color coordinates and fruit length, PAP for fruit weight, pericarp thickness and pH.

### Reference-guided, big variants discovery and de novo assembly of four pepper genomes

The iterative variant calling, and de novo assembly was used to generate two pepper genomes from Campania (CDT and PAP) and two from Calabria (SIG and CIL) regions. Using a total of 2.6 billion paired-end raw reads (780 Gb) (Table 2), we obtained a final genome size ~3Gb in each genome, which agrees with the estimated size of the pepper genomes available in literature [4, 5, 15]. The mapping rate was quite similar among the four accessions, ranging from 99.05% (PAP) to 99.3% (CIL) (Table 2). The average coverage depth varied from 19× in PAP to 26× in SIG, with a mean of 20× on almost all chromosomes (Table S1). The mapping coverage of these genotypes encompassed practically the whole length of the reference genome, except for some scaffolds (~ 1%), which showed a coverage less than 5×, therefore they were removed from the final genome assembly. All genomes were organized in the expected 12 chromosomes plus a “chromosome 0” obtained by concatenating the unanchored scaffolds, of which chromosome 1 resulted the longest in all genomes (288,705,451 bp on average) and chromosome 8 the shortest one (141,231,667 bp on average) (Table 3).

**Table 2:** Summary of results from sequencing data for each of the four genotypes (before and after the clean-up phase). The number of mapped and unmapped reads and the genome coverage results are also reported

Features	CDT	PAP	CIL	SIG
Number of cleaned reads	598,061,879	596,960,957	647,391,314	768,323,476
Mapped reads	593,360,465 (99.21%)	591,262,579 (99.05%)	642,828,463 (99.30%)	762,128,875 (99.19%)
Unmapped reads	4701 (0.79%)	5698 (0.95%)	4563 (0.70%)	6195 (0.81%)
Genome coverage	21×	19×	22×	26×
Mean Mapping Quality (Phred)	23.73	23.59	24.38	23.27
General error rate	0.76%	0.81%	0.68%	0.77%

**Table 3:** Chromosome size expressed as base pair (bp) in each re-constructed genome

Chromosome	CDT	PAP	CIL	SIG
chr1	290,784,941	281,071,636	303,750,627	279,214,599
chr2	167,325,089	161,873,474	163,955,390	162,820,872
chr3	275,838,308	271,864,947	243,386,756	273,128,636
chr4	230,594,104	229,879,515	231,656,043	230,301,839
chr5	226,647,541	141,981,671	213,706,438	224,613,793
chr6	226,420,338	223,600,010	226,729,294	220,890,558
chr7	241,523,990	240,232,178	242,424,026	241,732,915
chr8	141,198,383	140,962,733	141,311,165	141,454,387
chr9	263,499,724	257,641,826	264,936,163	263,991,586
chr10	222,324,002	230,003,064	226,500,929	224,778,264
chr11	260,196,731	257,143,330	264,408,616	260,006,969
chr12	237,943,671	226,513,104	246,276,889	234,865,442
chr0	152,636,143	152,048,786	156,905,955	156,718,321

After the first iteration of variant calling on CM334 reference genome, a total of 14,557,291 non redundant SNPs were identified among the four re-sequenced genotypes.

**Fig. 2:**
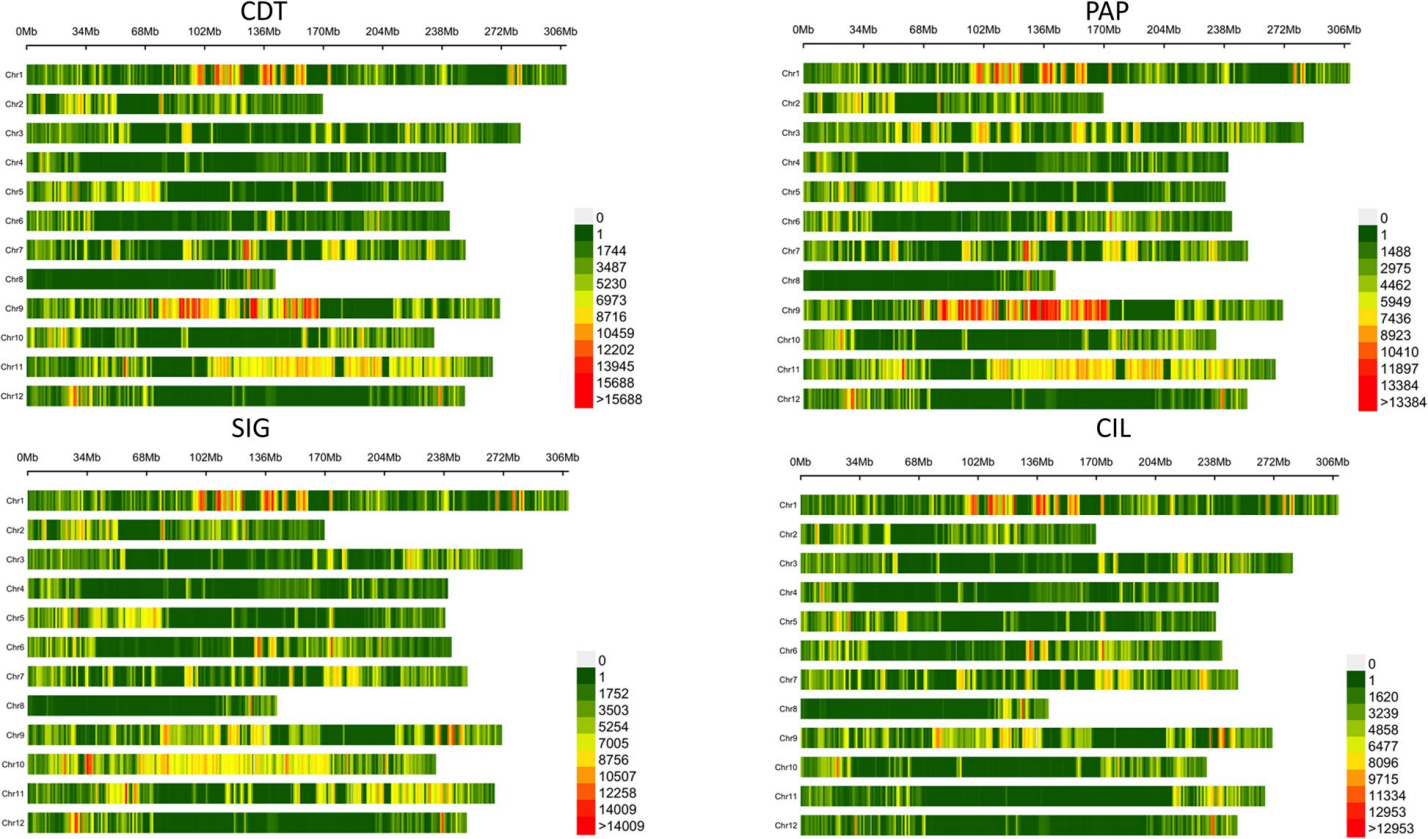
SNP density plot chromosome-wise for the four resequenced pepper genomes. The number of SNPs are represented within 1 Mb window size. The horizontal axis shows the chromosome (Chr) length (Mb); each bar represents a chromosome, with Chr 1 at the top and Chr 12 at the bottom.the different colors depict SNP density

The total number of polymorphisms was differently distributed and ranged from a minimum of 5,987,242 SNPs and 397,056 indels in CIL to a maximum of 8,233,170 SNPs and 535,349 InDels in SIG (Table S2). Overall, such variants were distributed among all chromosomes, with chromosome 8 showing the lowest number of polymorphisms (Fig. 2).

Following the sixth variant calling, Manta caller [18] was chosen for SV identification, since it is based either on discordant mapping signatures of paired reads or variations in read-depth. This approach allowed us to identify 15,986 SVs in the four pepper lines with respect to the reference. SVs were classified as deletions or PAV (*n* = 15,607), inversions (*n* = 154), and duplications or CNV (*n* = 225). Differences regarding SVs distribution among our samples were found (Table S2).

For example, a higher number of inversions were found on chromosome 11 in CDT and PAP but not in CIL and SIG. By contrast, four inversions were identified on chromosome 10 in SIG, whereas the other samples harbor at maximum one (Table S3). Similar to inversions, a higher number of deletions were found on chromosome 9 in CDT and PAP (Table S4), whereas no differences were identified for duplications, with the only exception for chromosome 6 with over three-folds duplications within Calabrian hot peppers with respect to sweet ones (Table S5).

De novo assembly of unmapped reads highlighted the highest diversity occurring within the considered genomes. In particular, we identified a total of 196 novel contigs, of which 24, 30, 33 and 36 were specific for CDT, PAP, CIL and SIG, respectively (Table S2; Fig. S2). Among all DNA sequences annotated in Solanaceae species (NBCI database March 6, 2017), all scaffolds showed high similarity with Zunla-1 genome. Ten scaffolds were in common to genomes from Calabria, whereas other three were in common to genomes from Campania (Fig. S2). Since all new scaffolds were not anchored to any chromosome, they were added to chrUn.

### Genome annotation

Prior to genome annotation, transcripts were assembled by reference guided with the Trinity pipeline [19] followed by filtering criteria based on the redundancy, abundance, and quality of the assembly to reduce the number of potential spurious contigs. Redundancy was eliminated by clustering our assembled transcripts with highly similar contigs using CD-HIT-EST (v4.6) [20] at a nucleotide identity of 95%. Fewer than 3% of the Trinity transcripts were redundant and were therefore removed. Expression levels were detected using KALLISTO [21] in order to remove transcripts not expressed. After this first filtering step, between 76 and 81% of the assembled transcripts were retrieved for each sample (data not shown). The number of final non-redundant transcripts considered for downstream analysis such as functional annotation, was roughly 13 k for each genome. Using this subset, together with transcripts from CM334 and Zunla1, we annotated on average 47 k genes per sample (Table 4).

**Table 4:** Number of predicted genes, mRNAs and proteins in each genome

Features	CDT	PAP	CIL	SIG
Predicted genes	47,996	46,096	47,663	48,389
mRNAs	52,082	49,684	50,546	52,238
Proteins	52,046	49,646	50,516	52,183
Longest proteins	47,998	46,102	47,662	48,378

In addition, we found about 10,000 genes not annotated in the reference genome although they were similar to those reported for other Solanaceae such as *Solanum tuberosum*, *S. pennellii* and *S. commersonii*. The lowest number of genes (Table 4) was detected in PAP (46,096), while the highest in SIG (48,389). Proteomes were validated using BUSCO and three different databases (*Eukaryote*, *Solanales* and *Viridiplantae*) (Table S6). Overall, more than 90% of expected proteins were identified as complete in all three databases. High percentage of putative paralogues (10 to 20%), i.e., complete genes with more than one copy were also observed. The function attributed to each predicted protein was based on the results of Gene Ontology (GO) and histograms with the abundances of GO terms were drawn using wego2 (https://wego.genomics.cn/) (Fig. S3). Among biological processes (BP), cellular and metabolic processes (accounting more than 60% of the predicted genes) were the most enriched terms in all genomes. Similarly, catalytic activity and binding (~ 60% of the predicted genes) abounded among the molecular functions (MF), whereas cell and membrane functions were the most abundant within cellular ones (CF) (60% of the predicted genes).

### Repeat proportion across pepper genomes

We also estimated the repeat proportion in our genomes through clustering analysis in RepeatExplorer2 [22]. The repetitive fractions of the genomes of all species were mainly composed of Long Terminal Repeats (LTRs), although a high proportion of these elements remained unclassified (~ 13%). Among the LTRs, the majority of elements belonged to *Gypsy* superfamily. In particular, ~ 8% were classified as *Tekay*, a well-known family of *Gypsy* chromovirus, whereas *Athila* and *Ogre* families abounded within Ty3_Gypsy category. By contrast, the most abundant elements belonging to *Copia* Superfamily were *TAR* and *Bianca.* To get more insights into the evolution of the repetitive fraction in our samples, a comparative analysis was also employed. Interestingly, we observed that the abundancy of clusters was different among our samples. For example, as shown in Fig. S4, CL195, CL108, CL105 and CL145, which together made the Supercluster 23, showed higher abundance in CIL compared with the others. Similarly, CL223 showed a similar size in SIG and PAP compared to the other samples, suggesting a specific burst of different families in each genome.

### Comparative analysis among pepper genomes

**Fig. 3:**
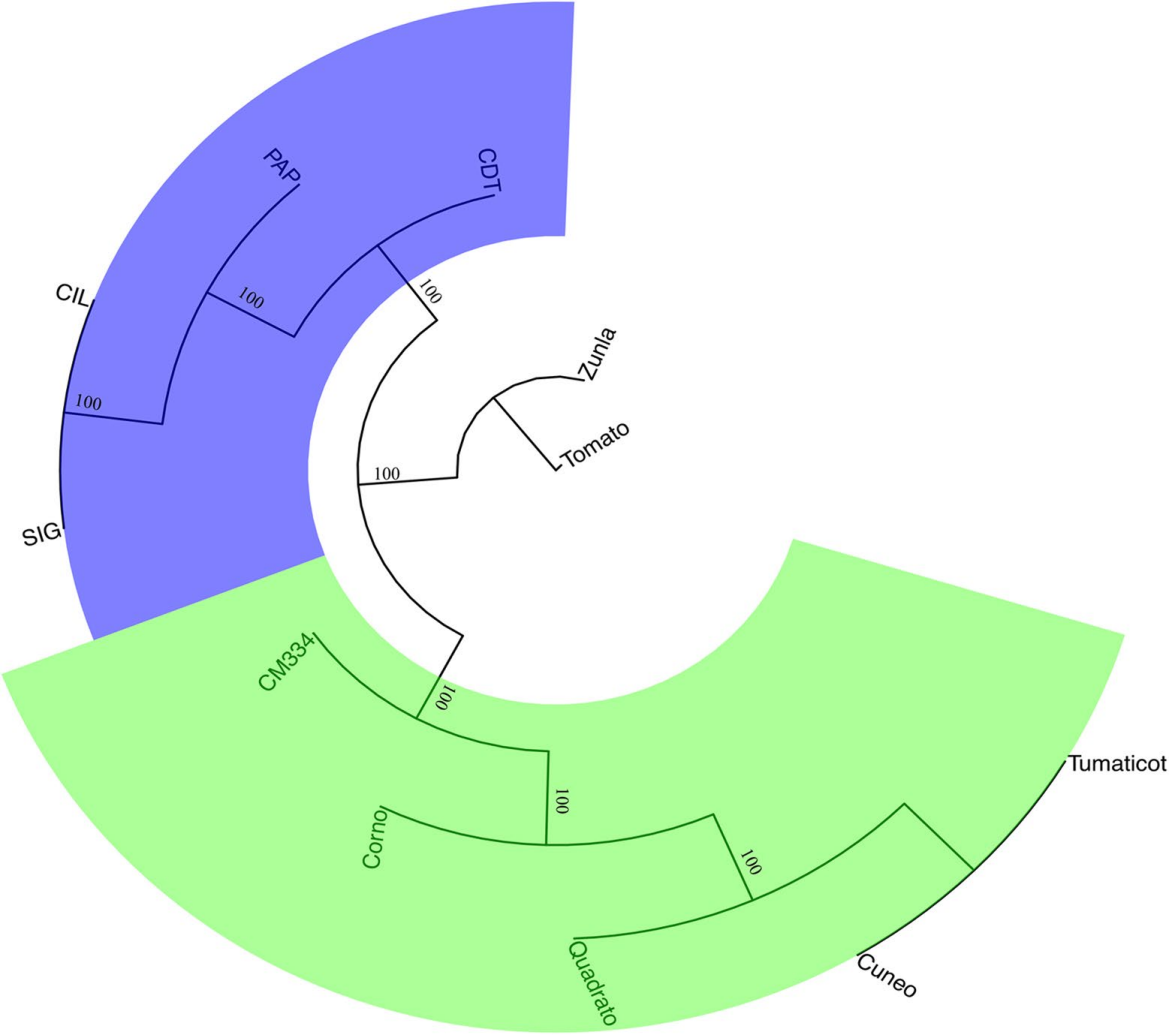
Clustering protein-based analysis. Clustering was carried out using the predicted proteins of the four pepper genotypes under study along with those analyzed by Acquadro et al. [15]. Bootstrap values of the consensus tree (out of 100) are given. Tomato was also included as an outgroup

The phylogenetic relationship inferred by annotated protein sequences of eleven proteomes with Orthofinder (169,465 sequences), including those reported in Acquadro et al. [15], the reference genomes ‘CM334’ and Zunla-1, and tomato as outgroup, are reported in Fig. 3. The analysis yielded a total of 49,475 gene families (plus 13,589 unassigned genes). Six-hundred eighty-five orthogroups were tomato specific and were not investigated, whereas 13,669 orthogroups (for a total of 195,300 genes) were shared among all samples including tomato. Seven thousand and seventy orthogroups were lacking in Zunla-1 proteome but not in ‘CM334’. By contrast, 3904 were found in Zunla-1 but not in the reference ‘CM334’, suggesting a high gene diversity among the genomes under study. The proteome of re-sequenced genomes highlighted 285 genome-specific orthogroups (118 genes) in the samples analyzed here, which were missing in those of Acquadro et al. [15] but not in the reference genomes (CM334 and/or Zunla-1).

By contrast, 172 orthogroups were found within the genomes from north Italy but not in those from south Italy. No specific orthogroups were found within yellow peppers and the red ones, suggesting that variants rather than the number of genes may reflect the differences between red and yellow peppers. One orthogroup (OG0045047, containing a gene annotated as *Iq-domain 31*) was found only in CDT and Corno, which share the same shape and weight. Similarly, three orthogroups (OG0047373, OG0047513 and OG0047776) were found only in Tumaticot and PAP. The common genes comprise a cell division ftsH protein, which is known to promote cell elongation division and a D-UF295 domain-containing protein. In addition, 14 orthogroups were found in the long-shaped and small size peppers ‘CM334’, Zunla-1 and SIG, although a more in-depth functional characterization of these genes is needed.

### Functional classification of the small variants

Estimation of variant effect prediction highlighted a high proportion of variants classified as “modifier” (~ 99%). This category included the variants located in intergenic or intronic regions, or affecting noncoding genes, indicating that there is no evidence of impact or that their predictions are difficult to assess (Table S7). The second most abundant variants impact class was “moderate” (0.30%) (i.e., nondisruptive variants, such as codon insertion/deletion or codon substitution) that might change protein functionality. The variants with “low” impact effects represented an average of 0.20% (ranging from 0.18% in CIL to 0.22% in SIG), whereas the “high” variation effects were less represented, being 0.03%. While the formers don’t cause changes in protein function (e.g., by mutating start and stop codons within the same amino acid), the latter’s are considered disruptive to proteins (e.g., causing truncation or loss of function due to exon deletion/insertion, mutations in the start or stop codons, splice site modification). Each genome harbored less than 3000 variants with high effect, impacting on a total of 2270 genes in CDT, 2150 in PAP, 2185 in SIG and 1940 in CIL. Out of these, 914 genes were shared by all genomes. By contrast, 209 genes were in common to the red genotypes (PAP, SIG and CIL) and 422 were unique to the yellow one (CDT). In addition, 182 genes were in common between chili pepper (SIG and CIL), whereas 279 were shared between the sweet ones (CDT and PAP). Among the latter, two transcription factors belonging to the *bHLH* family (*bHLH18,* ID: PHT68576.1 and *bHLH80,* ID: PHT71510.1) were identified.

### PCA, hierarchical clustering and heterozygosity highlighted geographical-based differences

**Fig. 4:**
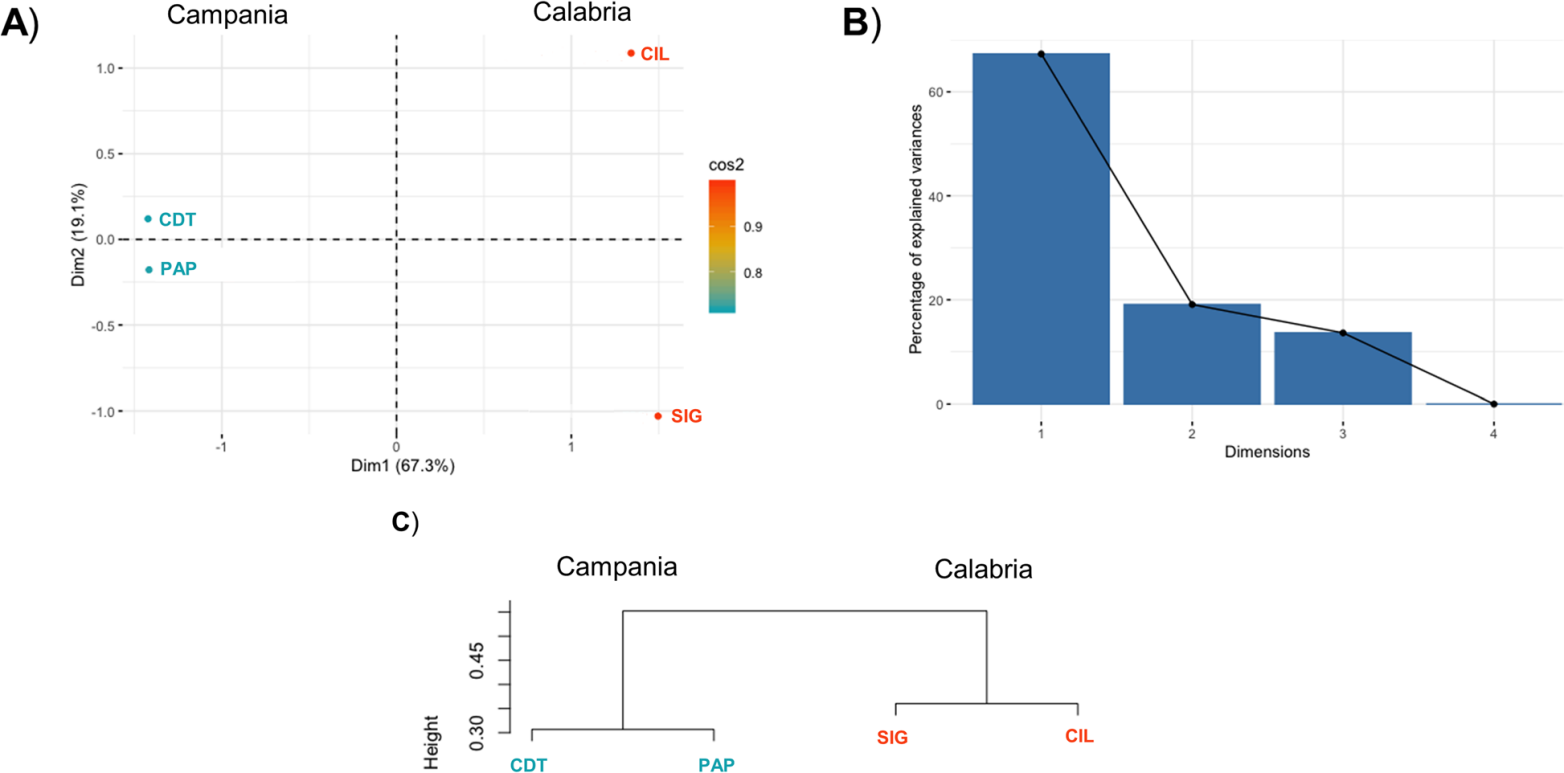
Genetic diversity among the four pepper genomes. **a** Loading plot of the first (Dim1) and second (Dim2) components; **b** Bar plot showing the percentage of explained variance of the first four components; **c** Hierarchical clustering of the four pepper genotypes

Substantial differences were observed among the four genomes based on geographical origin, with chromosome 9 and chromosome 11 showing more polymorphisms in genotypes retrieved from Campania region (Fig. 2). This observation was confirmed with Hierarchical clustering (Hclust) and principal component analysis (PCA), which revealed that genotypes can be clearly separated accordingly to their geographical origin, since just the first two components explained more than 80% of the total variance (Fig. 4).

**Fig. 5:**
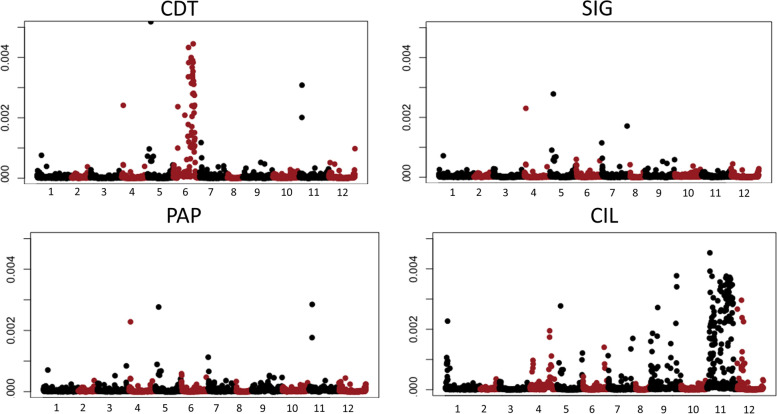
Heterozygous SNP density plots for the twelve chromosomes. The number of SNPs is within 1 Mb window size. The horizontal x-axis shows the twelve chromosomes, while the y-axis represents the number of heterozygous SNP divided per 1 Mb

Out of 29,258,818 SNPs identified within the four genotypes, heterozygous variants were analyzed in-depth. Interestingly, a peak of heterozygous variants was identified on chromosome 6 of CDT, whereas another peak was present on chromosome 11 of CIL (Fig. 5). By contrast, in PAP and SIG, heterozygous variants were less distributed along the chromosomes, with only few intervals showing a high number of heterozygotes. A GO enrichment analysis was then performed to identify significant terms harboring heterozygous variants among our genotypes. In CDT, three significant GO term (GO:0019001, GO:0032561 and GO:0002555) all involved in GTP functions were identified (Fig. S5). Among genes with these GO term, we identified a translation initiation factor (EIF2a), known to be involved in abiotic and biotic stress tolerance. By contrast, despite no significantly enriched terms were identified in CIL, interesting terms with regulatory functions of endogenous stimuli and biological quality were identified (data not shown).

### Private SNPs allowed the identification of variants associated to important agronomic traits

Both genomes from Campania were characterized by higher fruit weight and non-pungency of fruits, whereas those from Calabria had smaller and spicy fruits. For this reason, identifying private variants may allow to identify specific loci underlying these characteristics. Based on whole genome data, we identified 23,648 and 54,109 private variants in the genotypes from Campania and Calabria, respectively (data not shown). Although these variants were localized on all chromosomes, a large number of polymorphisms were found on chromosome 9 (1535 genes) in the former genotypes, and on chromosome 2 (1700 genes) and chromosome 3 (2896 genes) in the latters (Table S8). In addition, a higher density of private variants has been observed on chromosomes 7 and 9 (Fig. S6). Over 95% had a modifier effect in both groups, being 2294 out of 2402 and 9564 out of 10,057 presents in genomes from Campania and Calabria, respectively (Table S9). Interestingly, among variants with high impact in the genomes from Campania, we identified 4 polymorphisms localized in a gene annotated as *extension* (ID: PHT73052.1). In particular, the first variant was due to an insertion (T - > TA) in the genotypes from Campania with big and sweet fruits that induces a frameshift and, as consequence, the creation of a new stop codon (Fig. S7). By contrast, among variants with high impact in the genomes from Calabria, we identified two SNPs on chromosome 9 localized in two different genes belonging to the same gene family (*1-aminocyclopropane-1-carboxylate*, *ACO*).

Finally, we determined private variants in genes underlying the most important quality-related pathways in pepper (phenylpropanoids, carotenoids and flavonoids): six of them were involved in the phenylpropanoid pathway (*4-coumarate--CoA ligase 1*, *Caffeic acid 3-O-methyltransferase*, *Caffeoyl-CoA O-methyltransferase*, *Cytochrome 98A1*, *Oxalate--CoA ligase* and *Trans-cinnamate 4-monooxygenase*), five in flavonoids (*Chalcone-flavonone isomerase, Flavonol synthase/flavanone 3-hydroxylase, Chalcone synthase 2, Naringenin,2-oxoglutarate 3-dioxygenase* and *Leucoanthocyanidin dioxygenase*) and seven in carotenoids (*Capsanthin/capsorubin synthase*, *Zeta-carotene desaturase*, *Beta-carotene hydroxylase 1*, *Prolycopene isomerase*, *Lycopene epsilon cyclase*, *Protein Lutein Deficient 5* and *Carotenoid Cleavage Dioxygenase 4*) (Table S10, Table S11). For all candidates, at least one ortholog in each genome was identified, with the only exception for *capsanthin/capsorubin synthase,* which was not found in the yellow genotype CDT. The highest variability in terms of number of genes and/or isoforms was found for the *caffeic acid 3-O-methyltransferase*. Indeed, the highest number of genes and isoforms was found in CM334 and CIL, whereas the lowest was found in SIG (Table S10). Among these genes, two upstream variants were found in genomes from Campania affecting *caffeic acid 3-O-methyltransferase* and *putative chalcone-flavonone isomerase 3*. By contrast, upstream variants within *4-coumarate--CoA ligase 1* and *Pentatricopeptide repeat-containing protein,* and an intron variant in *Chalcone--flavonone isomerase* were identified in genomes from Calabria (data not shown). With the same approach we also investigated variants in 55 genes belonging to categories controlling berry size and shape. A variant localized in *brassinosteroid insensitive2-like2* (*BIN2-LIKE 2*) was found in both genomes from Campania, whereas three genes were affected by variants (*KLUH*, *Gigantea* and *AINTEGUMENTA*) in those from Calabria.

### Melting profile of *extensin*

**Fig. 6:**
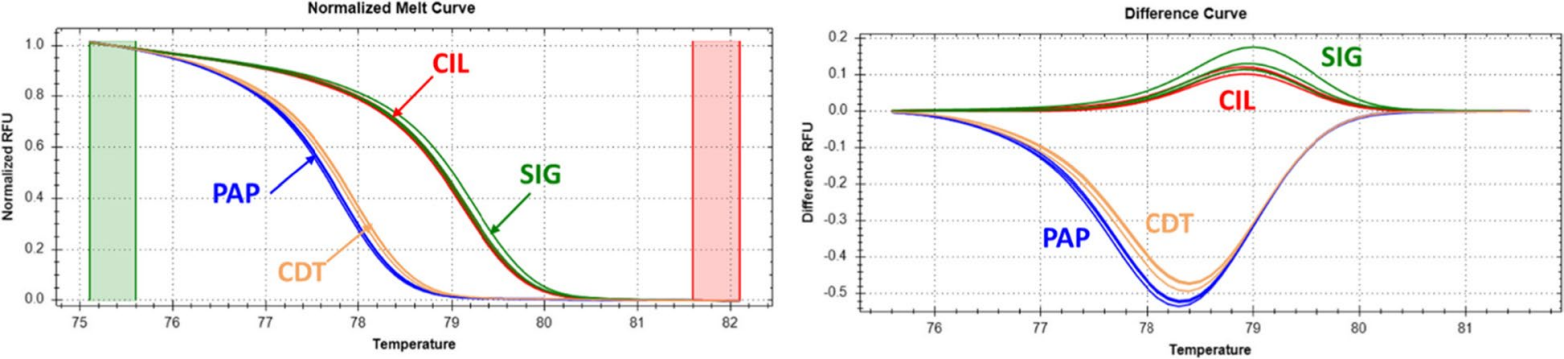
High resolution melting analysis. HRM profile of the 106 bp region delimiting a frameshift polymorphism found in the *extensin* gene. The left side is a melting curve analysis, and the right side is a difference plot graph

For high resolution melting analysis, a primer set was designed to amplify the region surrounding the frameshift mutation within the extensin sequence. For the best resolution, primers were designed to amplify the shortest possible region required for targeted genotyping [23]. The Ext marker produced two melting curves patterns in the tested accessions, confirming the polymorphism between the accessions from Campania and Calabria regions. Indeed, distinct melting curve patterns between CDT/PAP and SIG/CIL were observed (Fig. 6). In all triplicates, the frameshift mutation determined a difference of 1 °C of the melting temperatures between samples from Campania and Calabria, being 77.8 °C (CDT and PAP) and 79.1 °C, respectively. In CDT and PAP melting began at 73.4 °C and ended at 80.3 °C; in SIG and CIL melting started at 75.2 °C, whereas the ending T was 82.1 °C.

## Discussion

Next-generation sequencing (NGS) approaches for exploring crop genomes, open new opportunities for studying the genomic variation of plant species to be applied in plant breeding. Recent progress in NGS technologies and bioinformatics pipelines allow depth investigation of key regions and mechanisms underlying the variation of traits [24]. Nowadays, to avoid biased data interpretation and identify genome regions affected by genetic erosion, sequencing and assembly multiple reference genomes from crop wild relatives, local varieties and ecotypes are needed, as also emphasized by Aflitos et al. [25] for tomato wild relatives.

In this study, we performed a whole-genome resequencing analysis of pepper, in which four phenotypically diverse pepper materials from different Italian geographic regions were included, providing useful novelties that may have significant relevance for breeding of locally adapted varieties. Using the Illumina technology, we have mapped the over 1.2 billion paired-end high-quality reads of the four accessions against the reference genome “CM334” [4], obtaining a genome size of roughly 3.2 Gb for each sample, consistent with other pepper genomes [4, 5, 15]. Indeed, despite differences in the technology used, all *C. annuum* genomes released so far had a size ranging from 3 to 3.5 Gb. The genome size of the first sequenced and assembled pepper genome (*C. annuum* cv. CM334) was estimated at 3.48 Gb, similar to those of *C. annuum* Zunla-1 and of Chiltepin (*C. annuum var. glabriusculum*) released by Qin et al. in the same year. Our data are also consistent with results of resequencing efforts made with other *C. annuum* lines such as Dempsey (large bell-type genotype), Perennial (a genotype with small elongated fruits), Cuneo, Corno, Quadrato, Tumaticot, and the F_1_ hybrid from a cross between ‘CM334’ with a non-pungent blocky accession of *C. annuum* [4, 5]. The overall mapping coverage was around 20× and encompassed practically the whole length of the reference genome for all accessions. The high percentage of mapping rates of our samples (over 98%) confirmed the high-quality of the “CM334” reference genome, in line with other model species such as tomato [26, 27] and rice [11, 28]. Despite the low rate of unmapped reads, our de novo approach assembly of unmapped reads allowed us to disclose genomic regions and genes lacking in CM334 but not in Zunla-1. These findings suggest that the presence of a stretch of nucleotides into these DNA sequences may be the result of introgressions from Zunla-1 or that these sequences have been lost during CM334 assembly procedure. Most of these matches corresponded to annotated gene loci and therefore they could be of significance for the characteristic phenotypes of these genotypes.

As far as structural variation is concerned, their number and types were in general consistent with what has been reported in other plant species [29, 30], although the use of different methods and parameters in different studies make them difficult to compare. Indeed, it is difficult to compare our results with other pepper genomes, since in resequencing projects, SVs were usually not annotated and discussed. Our study has also identified one of the largest set of polymorphisms to date in pepper, consisting of almost 9 million of high-quality variants/sample, in line with other studies [15]. For example, 6 and 9 million SNPs were identified from wild and cultivated soybean accessions [11], 1.7 million from wild rice accessions [10], and over 10 million from wild tomatoes [23]. More than 90% of the polymorphisms were found in intergenic and noncoding intragenic regions, being the variants in coding regions < 9% on average, as also observed in other crops [12, 24, 31].

Interestingly, accessions from Campania and those from Calabria showed different genomic regions with a large density of SNPs (herein called private SNPs), which visually are like large blocks of peaks when the SNPs are divided and plotted into 1-Mbp sized bins. For example, we disclosed two SNPs with high impact on chromosome 9 localized in two paralogs of *ACO* genes. *ACO* is a member of the Fe II-dependent family of oxidases/oxygenases which require Fe^2+^ as a cofactor, ascorbate as a co-substrate and CO_2_ as an activator. This enzyme catalyzes the terminal step in the plant signaling of ethylene biosynthetic pathway and is known to be involved in the molecular mechanisms of climacteric fruit ripening. Although pepper fruit ripening is typically considered to be non-climacteric [32, 33], new clues pointed out that fruit ripening of hot pepper such as those retrieved from Calabria, is climacteric, with patterns of ethylene production during ripening [34]. These findings confirm the robustness of our SNPs, since sweet pepper such as those from Campania did not harbor the variants in *ACO* genes. On the contrary, two SNPs with high impact were identified in the genomes from Campania but not in those from Calabria. The two variants were identified in a gene called *extensin (EXTs). EXTs* are thought to act as self-assembling amphiphiles essential for cell-wall assembly and growth by cell extension and expansion [35, 36]. This is of a particular interest, since both genomes from Campania shared a high fruit weight, suggesting that this gene might be implicated in fruit extension. The analysis of the polymorphisms within *extensin* through high-resolution melting showed a clear differentiation between the big non-pungent fruits from Campania and the small pungent ones from Calabria. Anyhow, additional investigation of melting profile in a larger set with different fruit shapes did not confirm the differentiation between large and small-fruited accessions (data not shown). This is expected considering the quantitative nature of fruit shape [3, 37, 38], highlighting that there is no functional correlation between the mutation and fruit development, thus suggesting that this marker could be considered as specific only for fingerprinting of the investigated accessions.

We also identified a set of genes involved in phenylpropanoid, carotenoid and terpenoid pathways, and studied their variations among the four genotypes. Interestingly, a gene named *capsanthin/capsorubin synthase (CCS)* was lacking in the yellow genotype CDT, whereas the red genotypes harbor one copy each. Acquadro et al. [15] reported missing reads in the CCS gene surrounding the coding sequence and its promoter, that lead to a trunked protein in two of the yellow-fruited inbred lines (‘Cuneo’ and ‘Tumaticot’). However, this was not observed in all yellow genotypes, since the red fruited ‘Quadrato’ and the yellow-fruited ‘Corno’ showed a similar pattern. Thus, we hypothesize that the yellow color of CDT is related to the lack of *CCS* gene rather than differences in its promoter. In any case, a more in-depth functional characterization of these genes is needed. A previous study by Hill et al. [37] reported 17 private chromosomal blocks in non-pungent lines, underlying the regulation of organ size and capsaicin biosynthetic genes. Although we identified several SNPs in these regions, it was not possible to confirm these regions fixed only in the sweet accessions except for a 3969 bp region on chromosome 9 in the interval 4,192,541 bp to 4,196,510 bp. This region was located upstream of the gene *CA09g02160* which underlies organ growth, and size. The contrasting results could be due to the different cultivars used in the two studies and the different marker densities. Indeed, the higher SNP density used in the present study highlighted polymorphisms for pungent lines in these regions.

RNA-seq data from each sample were also produced to identify gene loci as well as derive transcript structural annotations within the reconstructed genomes. After removing redundant transcripts (i.e. duplicated and/or fragmented sequences) we annotated an average of 47 k genes per sample. This number includes about 10,000 genes not annotated in the reference genome and is similar to those reported for other *Solanaceae*. The higher number of annotated genes in our genotypes respect to ‘CM334’ could be explained either by an actual higher gene content of the two landraces or by a lack of gene annotations in the reference genome. It is also known that *C. annuum* underwent an expansion of the size of its genome caused mainly by the amplification of repetitive DNA sequences, including mobile genetic elements, as also recently reported by Yañez-Santos et al. [39]. Consistent with the information obtained by the latter authors, *Gypsy* superfamily were the most abundant TEs in our ecotypes. Such highest abundance has already been reported using different approaches in various *Solanaceae*, including the assembled genomes of tomato [40], *S. pennellii* [41], potato [42], *S. commersonii* [43, 44], and *S. chacoense* [45]. Although most of the evolutionary lineages of LTR retroelements described in Angiosperms were identified in pepper, three of them were the most abundant (*Athila*, *Ogre* and *Del*/*Tekay*). This result contrasts with the one observed in the genus *Solanum*, where a majority of *Del*/*Tekay* were detected. However, our results need to be confirmed by other approaches. It is noteworthy that differences in terms of supercluster abundancy were observed between the genotypes under study. This would indicate that a differential evolutionary dynamic would be shaping the composition of retroelements in their genomes. It is known that the amplification of certain types of repeats can occur rapidly within a species [46]. Thus, it is possible that such specific TE clusters underwent amplification at different periods and levels during the evolutionary process of our samples, as also showed by Huang et al. [47] and Baucom et al. [48]. It would be intriguing to test whether domestication processes underlie those differences.

## Conclusions

Changes in consumer’s preference and major attention to the conservation of biodiversity led to the rediscovery of landraces as source of novel variation to be used for both genetic improvement as well as for boosting microeconomies. Therefore, a deep characterization of the genetic variation is crucial toward full exploitation of such valuable germplasm. By whole genome resequencing we highlighted the high variation occurring in local varieties, reflecting both phenotypic as well as origin-based differences. This knowledge opens the frontiers to precision gene mapping, marker discovery, comparative studies. Indeed, the observed variation within key genes for quality pathway also allows precision breeding through future genome editing approaches. In addition, genome studies could enhance the procedure for the recognition of specificity local agri-food products as Protected Geographical Indication (PGI) and Protected Designation of Origin (PDO).

## Materials and methods

### Plant material and phenotypic characterization

Represented phenotypic data consider the average of two years of cultivation. Traits described include total yield (TY) based on the whole plot production (3 blocks/10 plants per block for each genotype), average fruit weight (FW) (in grams) obtained by weighting a set of 50 representative fruits, fruit length (FL) and fruit width (FD) (using a ruler), fruit shape index (FS) as length/width ratio, pericarp thickness measured by using manual calliper on ten fruits. On a bulk of 20 fruits color CIELAB coordinates (L*, a*, b*) and Chroma [(a*)^2^ + (b*)^2^]^0.5^ were measured by handheld colorimeter (Minolta Chroma Meter CR-210; Minolta Corp., Osaka, Japan). Soluble solids content (SSC) was measured by a digital refractometer (Refracto 30PX, Mettler-Toledo, Novate Milanese, Italy), and results were expressed as °Brix on 100 g of fresh weight (FW) [38]. The pH and the titratable acidity (TA) (expressed as g citric acid/L) juice were determined following the protocol described by Tripodi et al. [38]. Ascorbic acid (AsA, Vitamin C) and a-tocopherol (a-toc, Vitamin E), and Capsaicin (Caps) were measured by high-performance liquid chromatography (HPLC) following the protocol previously reported [38, 49].

### Statistical data analysis

Trait means and standard deviations were calculated in R version 3.5.1 [50]. Principal component analysis (PCA) was performed using the computer package XLSTAT 2012.1.

### Nucleic acid extraction

For each accession, genomic DNA was extracted from young leaves using the DNeasy® Plant Mini Kit (QIAGEN, Germany). DNA quality parameters as well as concentration were measured both by absorbance values at 260 nm and 280 nm, using a UV-Vis spectrophotometer (ND-1000; NanoDrop, Thermo Scientific, Wilmington, DE, USA) as well as using Qubit 2.0 Fluorometer based on Qubit dsDNA HS Assay (Thermo Fisher Scientific, Waltham, MA, USA).

RNA extraction was performed on mature fruits; tissues were ground into fine powder in liquid nitrogen, then, the RNA was isolated using the Norgen RNA Purification Kit (Norgen Biotek Corp, Ontario, Canada) following manufacturer’s instructions. RNA quality was checked by 1% (w/v) agarose gel electrophoresis, and its quantity, integrity and purity were assessed by Agilent 2100 Bioanalyzer (Agilent Technologies, Santa Clara, CA, USA).

### Illumina paired-end sequencing and reference-guided assembly

One μg of DNA was used for the construction of four Illumina DNA and four Illumina RNA libraries (Novogene, Hong Kong), which were sequenced using an Illumina Novaseq 6000 (Illumina Inc., San Diego, CA, USA) and paired-end chemistry (2 × 150 bp). Raw Illumina reads were processed with a in house made python script, which implemented quality of raw reads using FastQC [51], trimming with BBDuk [52] to remove reads with poor quality ends (Q < 35) and trim 5′ and 3′ -end bases and perform quality check on trimmed reads. Reference-guided assembly was performed using a two-step strategy: iterative read mapping and de novo assembly of unmapped reads following the approach defined by the Reconstructor pipeline. In the first step, high-quality reads were mapped on the reference genome CM334 v.2 [53] using minimap2 and duplicated reads were filtered out from resulted BAM files using Picard MarkDuplicates [54]. Platypus [55] was then employed for variant calling analysis using the following parameters: minReads = 10, trimReadFlank = 10, minMapQual = 30, minBaseQual = 30 and minPosterior = 30. Five iterations of mapping and variant calling were performed in order to identify sequence variations such as SNPs, deletion and insertion polymorphisms (DIPs). At each iteration, only the homozygous variants with a read number (NR) higher than 10 were kept. The identified polymorphisms were used to edit the pepper CM334 reference genome using bcftools consensus [56], thus obtaining cultivar specific sequences. By contrast, although heterozygous variants were not included in the new references, their distribution along chromosomes were analysed in 1-Mb windows to identify genomic regions with high frequency of heterozygous variants. At the same time, private variants (SNPs and Indels) among genomes from Campania and Calabria were identified using bcftools [56]. Structural variants (SVs) were analysed using Manta caller with default parameters [18]. The second step, based on the de novo assembly of the reads that did not map on the reference genome, was performed by using Soap De Novo v4.0.5 [57] setting k-mer sizes 25, maximum read length 150 nt and average insert size 300 nt. Resulting contigs were filtered for size. Raw data from ‘CM334’ were downloaded from NCBI (PRJNA223222).

### Annotation and functional analysis of polymorphic regions

All variants identified during the first iterative variant calling were functionally annotated respect to the genome annotation with SNPEff [58]. In addition, private variants were identified in genomes from Calabria and Campania using bcftools [56] with -x option. Gene Ontology Enrichment Analysis (GOEA) was performed on the genes showing missense mutations as well as on genes with polymorphisms altering CDS length (i.e., disruptive in-frame deletions, disruptive in-frame insertions, frameshift variants, stop coding gain/loss and start codon loss). GOEA was performed with in-house scripts as described by Tranchida-Lombardo et al. [26].

### Genome and transposable elements masking

Before the annotation process, transcripts in each genome were assembled from custom RNA-seq data. Raw reads were trimmed with BBDuk (v. 0.33) [52] and mapped versus the reference genome with STAR (v. 2.4.2a) [59] with default parameters, except for “--twopassMode Basic --alignIntronMax 50000”. Mapped reads along with FASTA reference were subjected to Trinity (v2.0.6) [60] for reference-guided assembly. Then, three different filtering steps were performed: CD-HIT-EST tool [18] which clusters similar transcripts based on a similarity threshold (95% of identity), Kallisto to remove sequences that did not show expression and Blastx (v2.2.30) against a known protein database retrieved from NCBI. Following these steps, on average, 13 k sequences were given as input for gene prediction using Maker-P pipeline [61] to perform both a genome-guided and ab initio gene prediction. Augustus v3.3.2 [62] and SNAP [63] gene prediction algorithms were combined to support the predictions. All predicted gene models were filtered based on AED values ≤0.35. The gene function was assigned to each predicted gene by using Pannzer2 [64] with the following criteria: minimum query coverage and minimum sbcjt coverage = 0.4, and miminum alignment length set at 50. Benchmarking Universal Single-Copy Orthologs (BUSCO89 v3.0.2., *Viridiplantaedb10*, *Solanaceaedb10* and *Eukaryotesdb10*) was implemented to measure the quality and completeness of the predicted proteomes. Once the genome annotation was completed, we implemented ORTHOFINDER [65] with the following parameters: -M msa -A mafft -T raxml -ng to define orthogroups within our genomes and those re-sequenced by Acquadro et al. [15]. The evolutionary history was inferred using the Neighbor-Joining method [66]. The bootstrap consensus tree inferred from 100 replicates [67] is taken to represent the evolutionary history of the taxa analyzed [68]. Branches corresponding to partitions reproduced in less than 50% bootstrap replicates are collapsed. The evolutionary distances were computed using the JTT matrix-based method [69] and are in the units of the number of amino acid substitutions per site. All ambiguous positions were removed for each sequence pair (pairwise deletion option). Evolutionary analyses were conducted in MEGA11 [70]. Genes involved in phenylpropanoids, terpenoids and carotenoids were identified as described by previously [65, 71]. The repeated fraction was also evaluated by graph-based clustering of repetitive elements in unassembled reads using the Repeat Explorer2 Web server [22]. In detail, 500.000 random reads were filtered out by quality and used as input.

### High resolution melting analysis

Primer pairs surrounding the frameshift region (T > TA) within the extensin gene were designed using Primer 3.0. Not labelled Forward (ExtF: CGATAGCACCACCCTAACCT) and reverse (ExtR: TGGCAGGTATAAAGCAAGGC) allowed a single DNA amplified product of 106 bp (Fig. S7) For each HRM assay, three replicates were considered. PCRs have been performed in a volume of 10 μL per reaction in a 96-well Bio-Rad CFX 96 RealTime PCR System (Bio-Rad, Inc., Hercules, CA). Reaction mixtures included 5 μl of 2× Precision Melt Supermix containing hot start iTaqTM, DNA polymerase, dNTPs, MgCl_2_, EvaGreen dye (Bio-Rad) (final concentration 1×), 200 nM of each primer and 2.5 μL (conc 15 ng/μL) of genomic DNA of the four resequenced accessions. Sterile H_2_O to final 10 volume. A negative control was included in each assay. Amplification consisted of an initial step at 95 °C for 2 min followed by 40 cycles of 95 °C for 15 s and 55 °C for 30 s. Then the melting analysis was performed following the protocol previously described [72].

## Supplementary Information

**Additional file 1: Figure S1.** Traditional varieties considered in the present study and their provenance. CDT = Corno di toro; PAP = Papaccella; SIG = Sigaretta; CIL = Ciliegino.

**Additional file 2: Figure S2.** Number of de novo assembled scaffolds in CDT, PAP, SIG and CIL, respectively. The number of common scaffolds is also shown.

**Additional file 3: Figure S3.** Gene Ontology (GO) classification using Web Gene Ontology Annotation Plot (WEGO) in four pepper genomes. The results are summarized in three main GO categories: cellular component, molecular function and biological process. The right y-axis indicates the number of genes in each category. The y-axis indicates the percentage of a specific category of genes in that category. One EST could be annotated into more than one GO term.

**Additional file 4: Figure S4.** Comparative analysis summary using RepeatExplorer2. Bar plot shows the sizes (numbers of reads) of individual top clusters. Rectangle size is proportional to the number of reads in a cluster for each genome.

**Additional file 5: Figure S5.** GO enrichment analysis of heterozygous variants identified in CDT. Each box shows the GO term number, the *p*-value in parentheses, and GO term. Box colors indicate levels of statistical significance.

**Additional file 6: Figure S6.** Physical localization of private SNPs identified on chromosomes 9 and 7 in genomes from Campania and Calabria. The strongest differences are marked with a blue box. The number of private variants per 1 Mb windows is reported on the y-axis.

**Additional file 7: Figure S7.** Extensin gene region (9:232692750-232694483_ID: PHT73052.1). Highlighted in yellow the mutation site (Insertion of adenine T - > TA in genotypes from Campania). Flanking primers are highlighted in red (ExtF) and blue (reverse complement of ExtR) font, respectively.

**Additional file 8: Table S1.** Mean coverage of each chromosome in all genomes analyzed.

**Additional file 9: Table S2.** Number of small (SNPs and INDELS) and big variants (Deletions, Duplications and Inversions). The number of new contigs is also shown.

**Additional file 10: Table S3.** Number of inversions per chromosome identified in the four genomes investigated.

**Additional file 11: Table S4.** Number of deletions per chromosome identified in the four genomes investigated.

**Additional file 12: Table S5.** Number of duplications per chromosome identified in the four genomes investigated.

**Additional file 13: Table S6.** BUSCO statistics for the four pepper genomes using *Eukaryote, Solanales* and *Viridiplantae* databases*.*

**Additional file 14: Table S7.** Statistics of SNPs/indels effects within each of the four genotypes. All variants (heterozygous and homozygous) are reported.

**Additional file 15: Table S8.** Number of genes showing private SNPs/Indels in genomes from Campania and Calabria, respectively.

**Additional file 16: Table S9.** SNPeff statistics regarding private SNPs in genomes from Campania and Calabria, respectively.

**Additional file 17: Table S10.** Gene and isoforms in the four heirlooms and the reference CM334.

**Additional file 18: Table S11.** Gene ID in the four heirlooms and the reference CM334.

## Data Availability

All sequence raw data of the reconstructed genomes along with their annotations (gff3) are available on figshare (10.6084/m9.figshare.15097038.v1).

## Abbreviations

TE: Transposable elements
LTRs: Long terminal repeats
SV: Structural variants
PAV: Presence-absence variation
CNV: Copy number variation
GO: Gene Ontology
BP: Biological processes
MF: Molecular functions
CG: Cellular functions
