# Supplementary material for: Whole-genome resequencing reveals genomic footprints of Italian sweet and hot pepper heirlooms giving insight into genes underlying key agronomic and qualitative traits
Source: BMC Genom Data. 2022 Mar 25;23:21. doi: 10.1186/s12863-022-01039-9 (PMC8957157; doi:10.1186/s12863-022-01039-9)
Supplement: Supplementary file 1 — Additional file 1: Figure S1. Traditional varieties considered in the present study and their provenance. CDT = Corno di toro; PAP = Papaccella; SIG = Sigaretta; CIL = Ciliegino. [file 12863_2022_1039_MOESM1_ESM.pptx]

## Slide 1
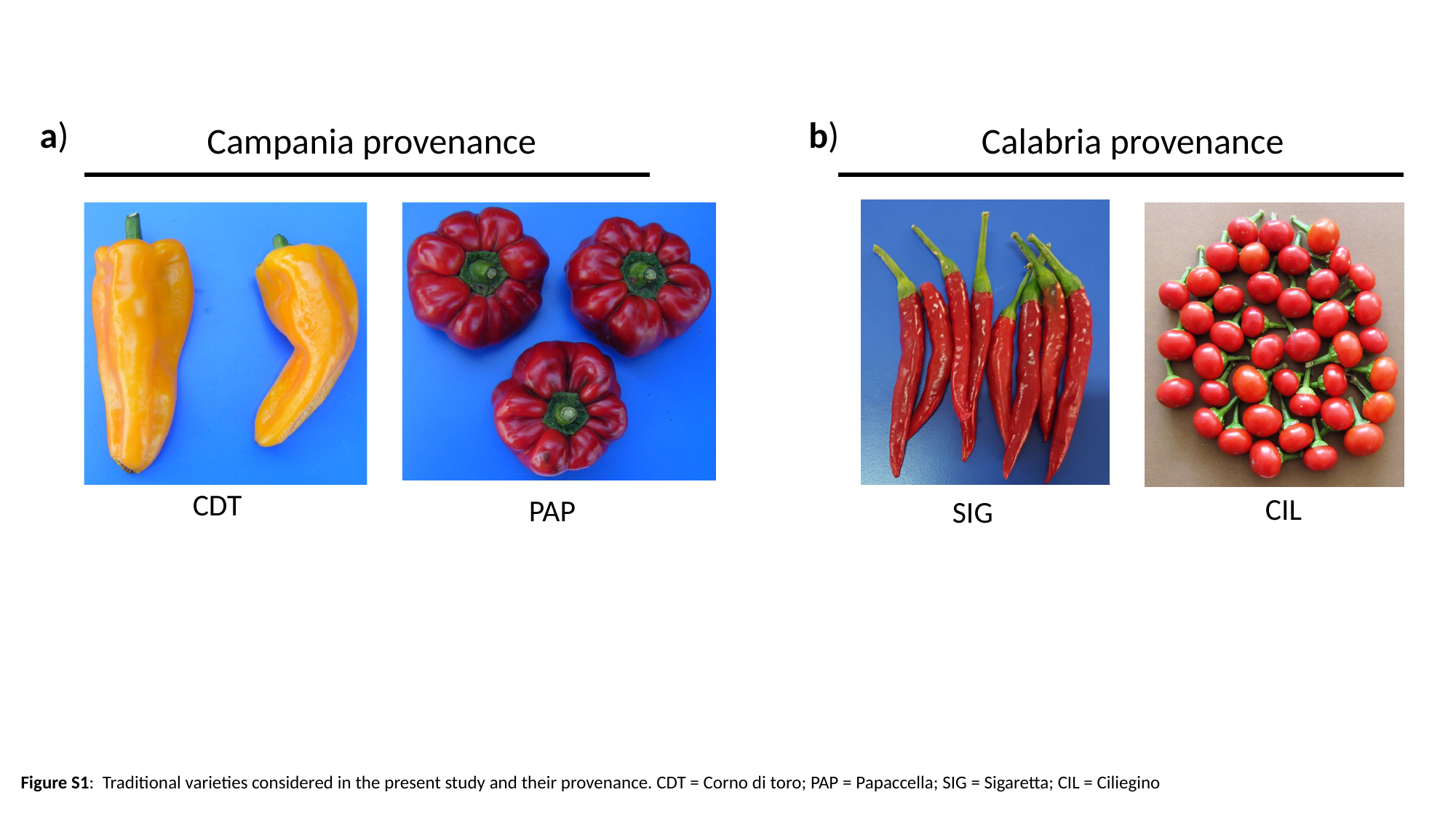

a)
b)
Campania provenance
Calabria provenance
CDT
CIL
PAP
SIG
Figure S1: Traditional varieties considered in the present study and their provenance. CDT = Corno di toro; PAP = Papaccella; SIG = Sigaretta; CIL = Ciliegino
