# Supplementary material for: Whole-genome resequencing reveals genomic footprints of Italian sweet and hot pepper heirlooms giving insight into genes underlying key agronomic and qualitative traits
Source: BMC Genom Data. 2022 Mar 25;23:21. doi: 10.1186/s12863-022-01039-9 (PMC8957157; doi:10.1186/s12863-022-01039-9)
Supplement: Supplementary file 2 — Additional file 2: Figure S2. Number of de novo assembled scaffolds in CDT, PAP, SIG and CIL, respectively. The number of common scaffolds is also shown. [file 12863_2022_1039_MOESM2_ESM.pptx]

## Slide 1
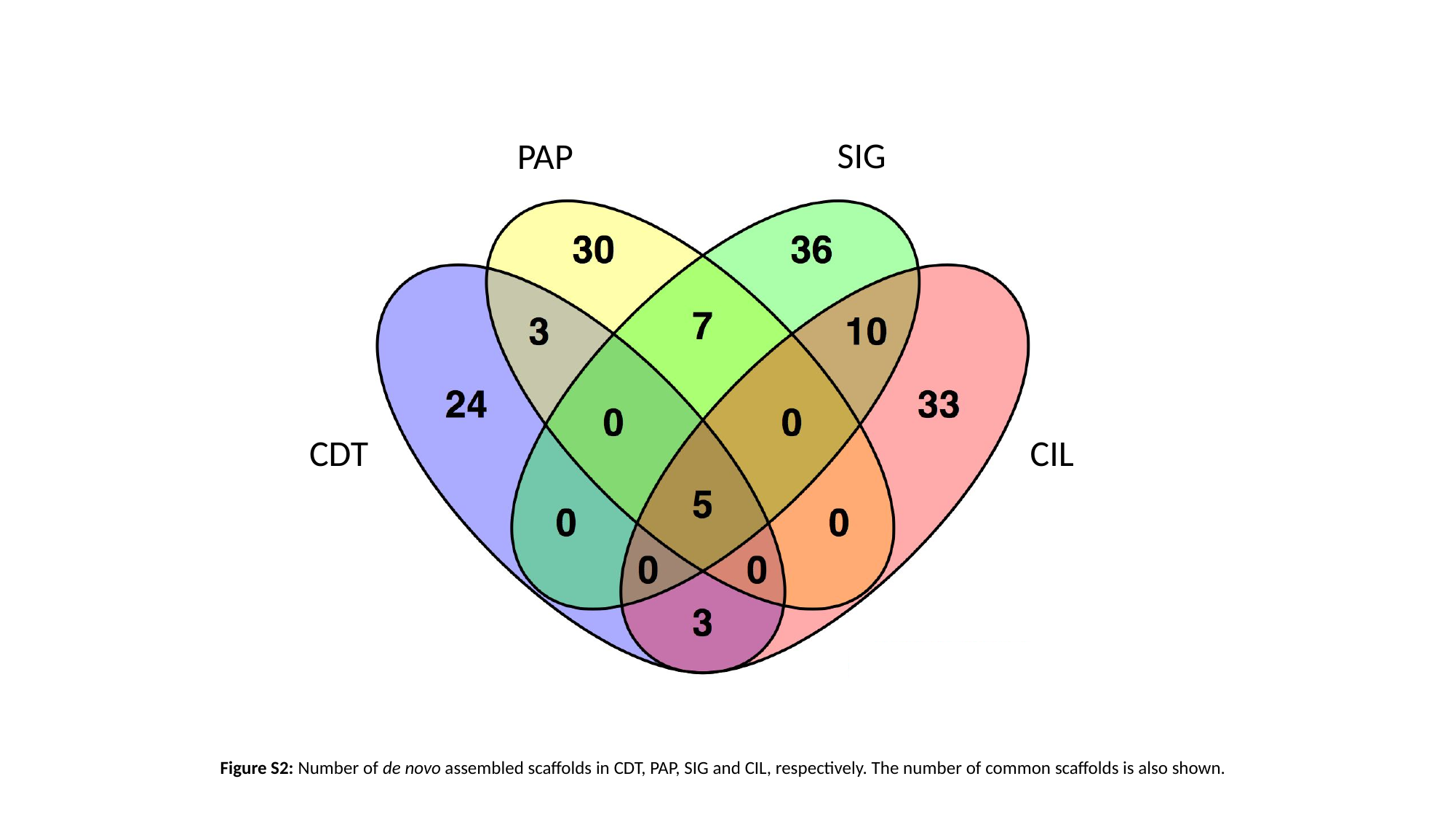

SIG
PAP
CDT
CIL
Figure S2: Number of de novo assembled scaffolds in CDT, PAP, SIG and CIL, respectively. The number of common scaffolds is also shown.
