# Supplementary material for: Whole-genome resequencing reveals genomic footprints of Italian sweet and hot pepper heirlooms giving insight into genes underlying key agronomic and qualitative traits
Source: BMC Genom Data. 2022 Mar 25;23:21. doi: 10.1186/s12863-022-01039-9 (PMC8957157; doi:10.1186/s12863-022-01039-9)
Supplement: Supplementary file 3 — Additional file 3: Figure S3. Gene Ontology (GO) classification using Web Gene Ontology Annotation Plot (WEGO) in four pepper genomes. The results are summarized in three main GO categories: cellular component, molecular function and biological process. The right y-axis indicates the number of genes in each category. The y-axis indicates the percentage of a specific category of genes in that category. One EST could be annotated into more than one GO term. [file 12863_2022_1039_MOESM3_ESM.pdf]

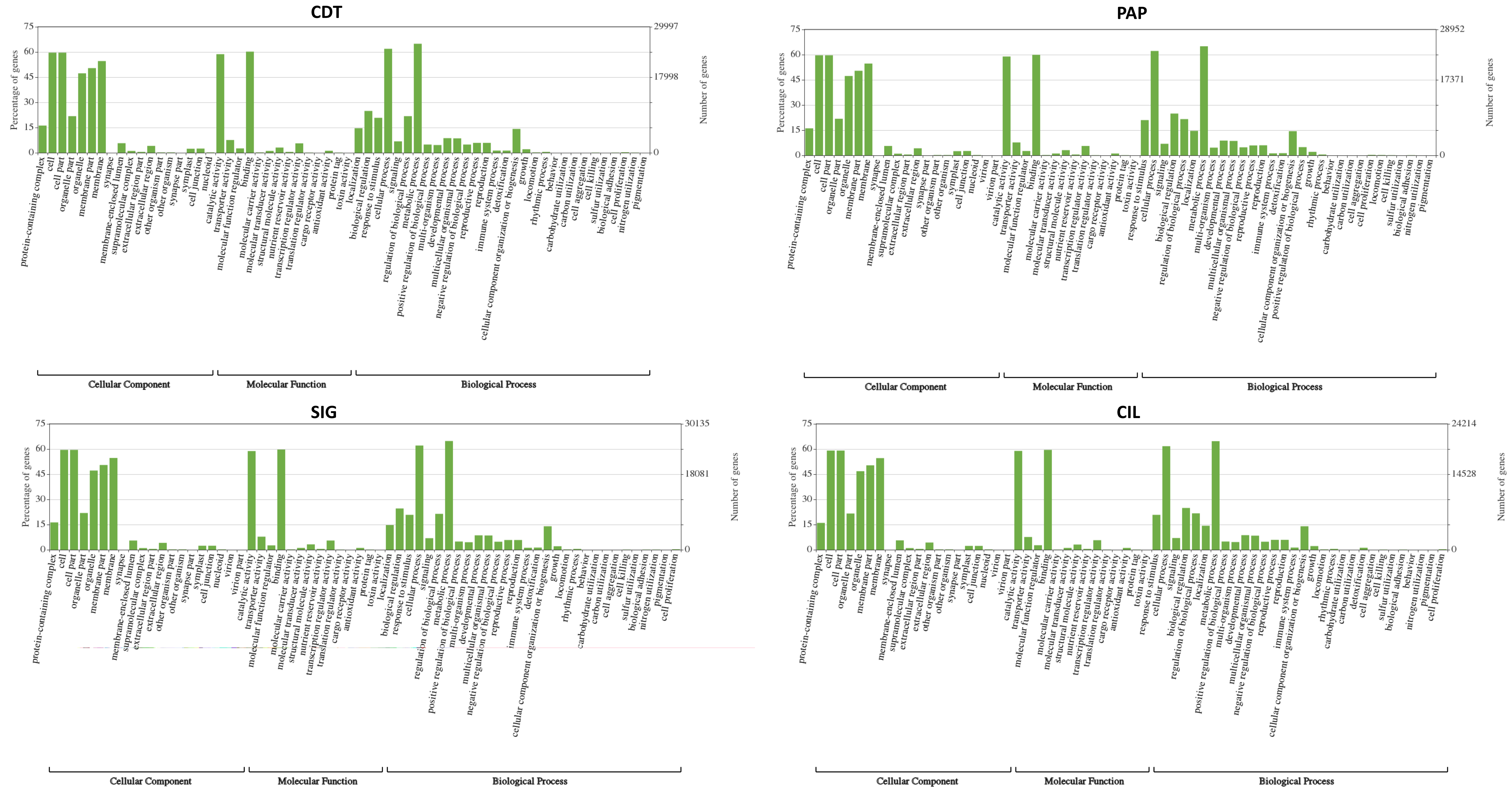

Figure S3: Gene Ontology (GO) classification using Web Gene Ontology Annotation Plot (WEGO) in four pepper genomes. The results are summarized in three main GO categories: cellular component, molecular function and biological process. The right y-axis indicates the number of genes in each category. The y-axis indicates the percentage of a specific category of genes in that category. One EST could be annotated into more than one GO term.
