# Supplementary material for: Whole-genome resequencing reveals genomic footprints of Italian sweet and hot pepper heirlooms giving insight into genes underlying key agronomic and qualitative traits
Source: BMC Genom Data. 2022 Mar 25;23:21. doi: 10.1186/s12863-022-01039-9 (PMC8957157; doi:10.1186/s12863-022-01039-9)
Supplement: Supplementary file 4 — Additional file 4: Figure S4. Comparative analysis summary using RepeatExplorer2. Bar plot shows the sizes (numbers of reads) of individual top clusters. Rectangle size is proportional to the number of reads in a cluster for each genome. [file 12863_2022_1039_MOESM4_ESM.pptx]

## Slide 1
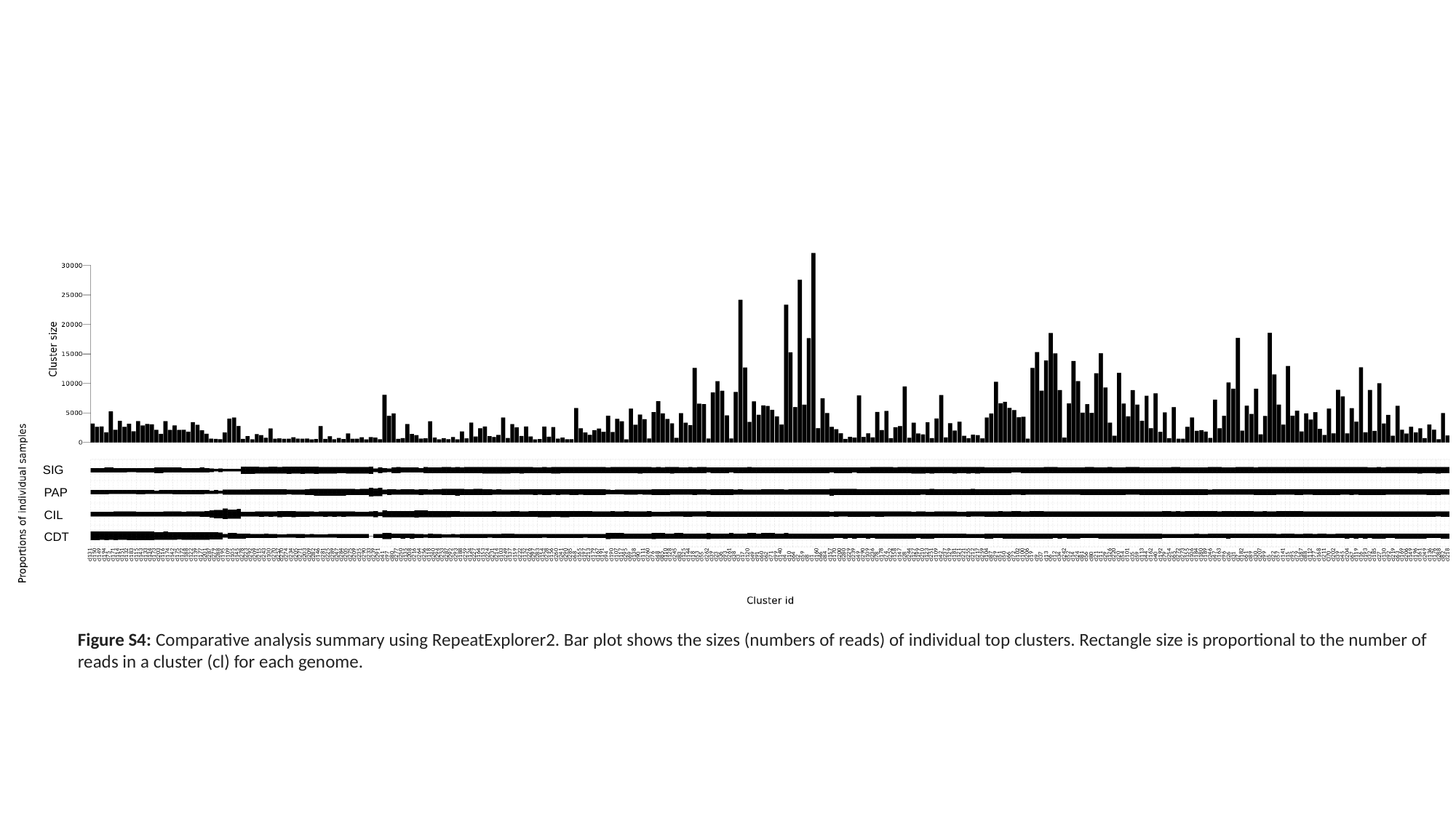

SIG
PAP
CIL
CDT
cl131
cl150
cl149
cl194
cl171
cl114
cl151
cl132
cl183
cl115
cl143
cl133
cl134
cl173
cl203
cl116
cl174
cl142
cl175
cl172
cl188
cl122
cl139
cl177
cl201
cl287
cl279
cl288
cl195
cl109
cl105
cl145
cl278
cl223
cl292
cl209
cl215
cl243
cl100
cl270
cl257
cl270
cl274
cl234
cl205
cl271
cl203
cl209
cl282
cl146
cl281
cl225
cl289
cl247
cl284
cl205
cl275
cl209
cl235
cl290
cl233
cl239
cl291
cl277
cl250
cl135
cl208
cl216
cl264
cl256
cl118
cl240
cl294
cl253
cl287
cl292
cl293
cl188
cl259
cl124
cl227
cl164
cl153
cl224
cl231
cl214
cl103
cl249
cl137
cl159
cl222
cl152
cl226
cl297
cl283
cl154
cl228
cl156
cl260
cl241
cl290
cl285
cl165
cl197
cl213
cl179
cl167
cl191
cl190
cl107
cl117
cl295
cl138
cl111
cl260
cl110
cl128
cl245
cl125
cl144
cl262
cl261
cl120
cl140
cl160
cl157
cl170
cl200
cl280
cl229
cl239
cl290
cl109
cl236
cl178
cl252
cl158
cl147
cl244
cl127
cl199
cl210
cl123
cl251
cl109
cl237
cl129
cl181
cl121
cl221
cl255
cl212
cl217
cl259
cl104
cl102
cl100
cl206
cl242
cl126
cl220
cl101
cl113
cl162
cl192
cl254
cl272
cl273
cl155
cl106
cl184
cl180
cl189
cl246
cl163
cl182
cl207
cl141
cl187
cl112
cl168
cl211
cl202
cl204
cl119
cl193
cl185
cl130
cl219
cl169
cl206
cl149
cl196
cl161
cl249
cl136
cl176
cl288
cl218
Cl70
cl75
cl41
cl97
cl80
cl68
cl94
cl69
cl90
cl76
cl48
cl85
cl83
cl18
cl53
cl55
cl39
cl23
cl36
cl93
cl38
cl17
cl49
cl91
cl60
cl62
cl71
cl99
cl10
cl64
cl59
cl46
cl84
cl49
cl78
cl74
cl28
cl42
cl87
cl29
cl51
cl50
cl66
cl72
cl19
cl37
cl13
cl12
cl34
cl52
cl14
cl24
cl81
cl56
cl80
cl21
cl11
cl29
cl20
cl54
cl35
cl59
cl44
cl40
cl79
cl65
cl47
cl96
cl26
cl31
cl61
cl89
cl30
cl99
cl22
cl57
cl15
cl95
cl79
cl88
cl77
cl39
cl45
cl67
cl16
cl32
cl27
cl92
cl63
cl82
cl3
cl4
cl2
cl8
cl1
cl9
cl6
cl7
cl5
Figure S4: Comparative analysis summary using RepeatExplorer2. Bar plot shows the sizes (numbers of reads) of individual top clusters. Rectangle size is proportional to the number of reads in a cluster (cl) for each genome.
