# Supplementary material for: Whole-genome resequencing reveals genomic footprints of Italian sweet and hot pepper heirlooms giving insight into genes underlying key agronomic and qualitative traits
Source: BMC Genom Data. 2022 Mar 25;23:21. doi: 10.1186/s12863-022-01039-9 (PMC8957157; doi:10.1186/s12863-022-01039-9)
Supplement: Supplementary file 5 — Additional file 5: Figure S5. GO enrichment analysis of heterozygous variants identified in CDT. Each box shows the GO term number, the p-value in parentheses, and GO term. Box colors indicate levels of statistical significance. [file 12863_2022_1039_MOESM5_ESM.pptx]

## Slide 1
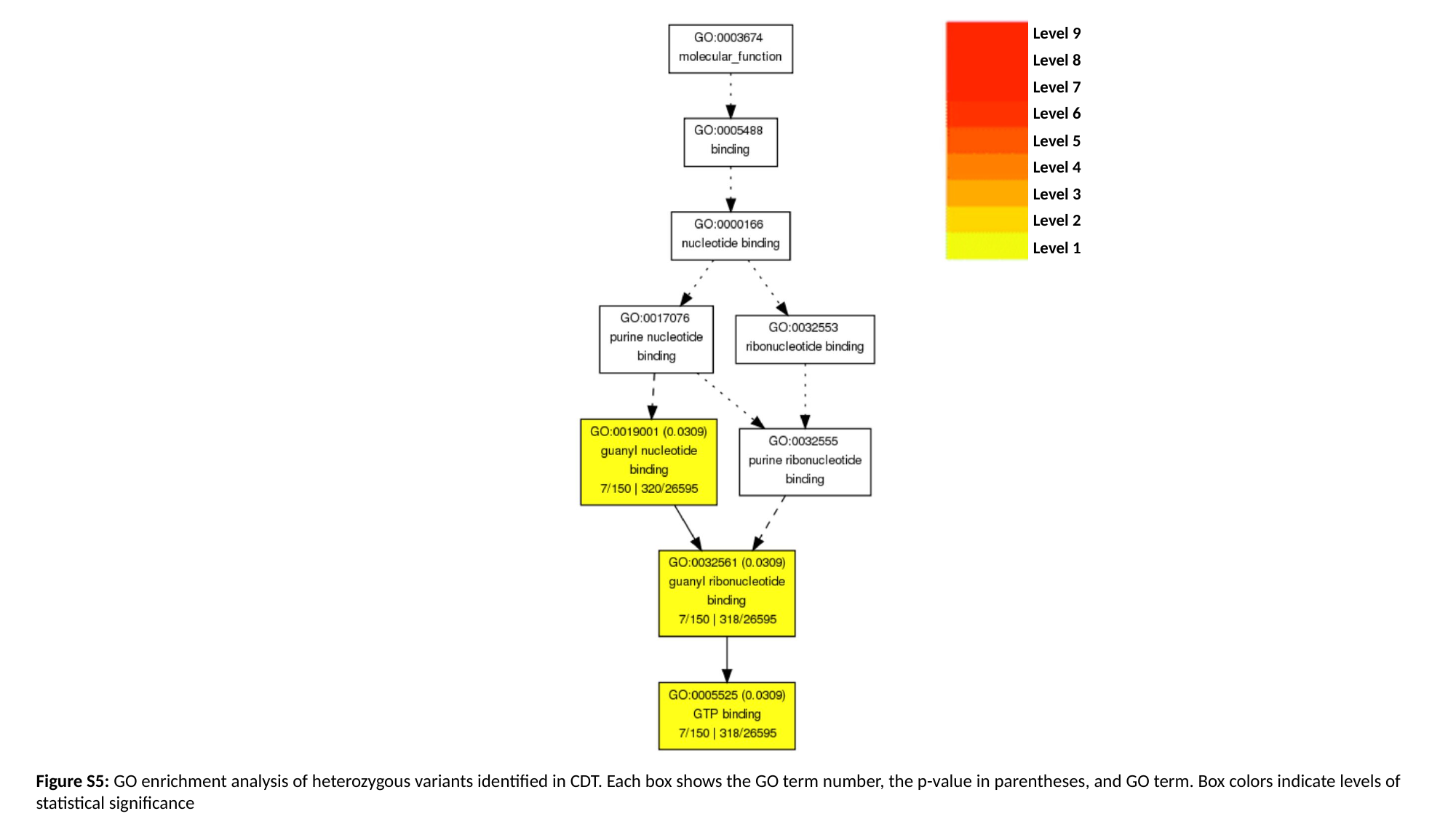

Level 9
Level 8
Level 7
Level 6
Level 5
Level 4
Level 3
Level 2
Level 1
Figure S5: GO enrichment analysis of heterozygous variants identified in CDT. Each box shows the GO term number, the p-value in parentheses, and GO term. Box colors indicate levels of statistical significance
