# Supplementary material for: Whole-genome resequencing reveals genomic footprints of Italian sweet and hot pepper heirlooms giving insight into genes underlying key agronomic and qualitative traits
Source: BMC Genom Data. 2022 Mar 25;23:21. doi: 10.1186/s12863-022-01039-9 (PMC8957157; doi:10.1186/s12863-022-01039-9)
Supplement: Supplementary file 6 — Additional file 6: Figure S6. Physical localization of private SNPs identified on chromosomes 9 and 7 in genomes from Campania and Calabria. The strongest differences are marked with a blue box. The number of private variants per 1 Mb windows is reported on the y-axis. [file 12863_2022_1039_MOESM6_ESM.pptx]

## Slide 1
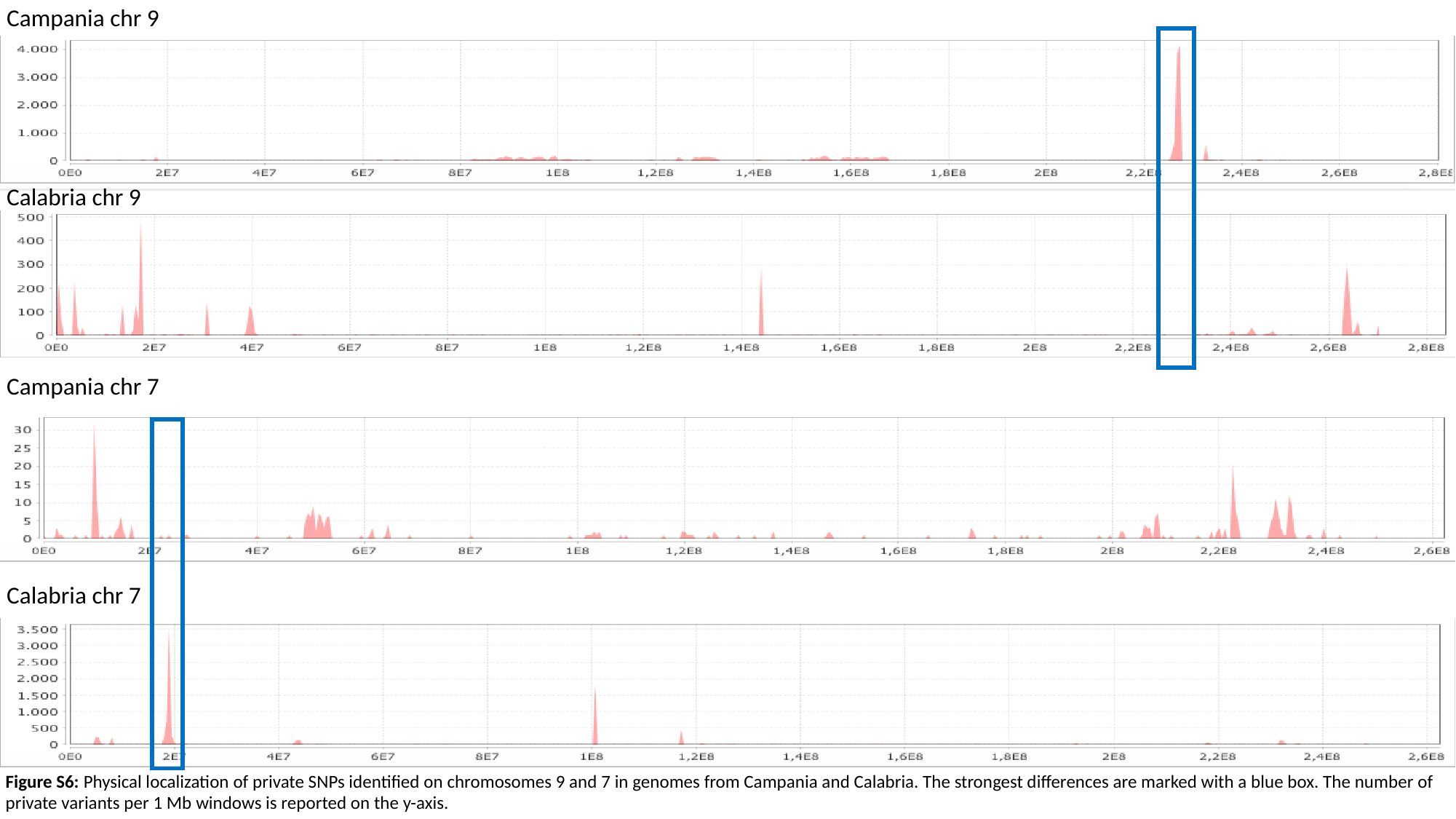

Campania chr 9
Calabria chr 9
Campania chr 7
Calabria chr 7
Figure S6: Physical localization of private SNPs identified on chromosomes 9 and 7 in genomes from Campania and Calabria. The strongest differences are marked with a blue box. The number of private variants per 1 Mb windows is reported on the y-axis.
