# Supplementary material for: Whole-genome resequencing reveals genomic footprints of Italian sweet and hot pepper heirlooms giving insight into genes underlying key agronomic and qualitative traits
Source: BMC Genom Data. 2022 Mar 25;23:21. doi: 10.1186/s12863-022-01039-9 (PMC8957157; doi:10.1186/s12863-022-01039-9)
Supplement: Supplementary file 7 — Additional file 7: Figure S7. Extensin gene region (9:232692750-232694483_ID: PHT73052.1). Highlighted in yellow the mutation site (Insertion of adenine T - > TA in genotypes from Campania). Flanking primers are highlighted in red (ExtF) and blue (reverse complement of ExtR) font, respectively. [file 12863_2022_1039_MOESM7_ESM.pptx]

## Slide 1
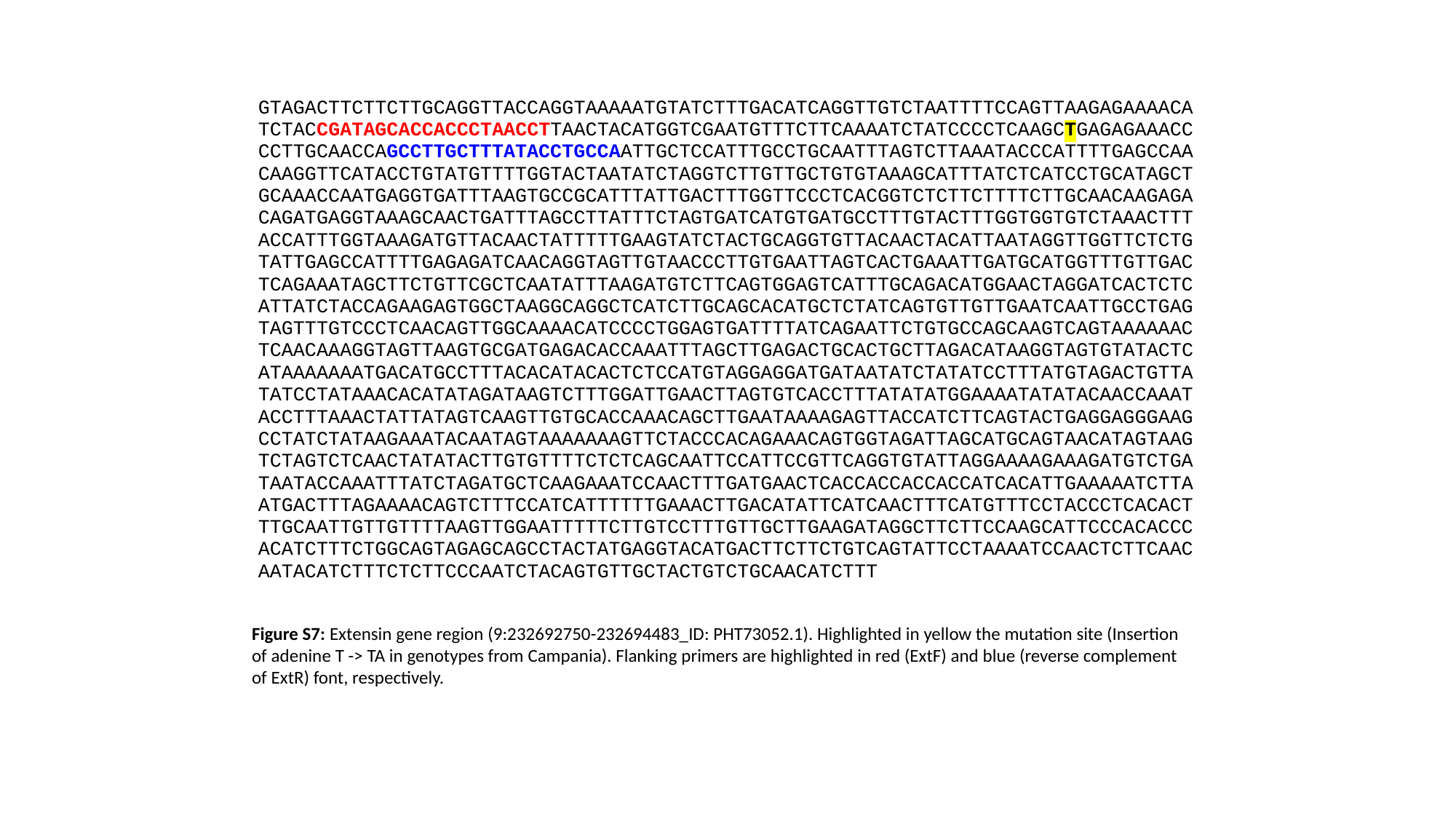

Figure S7: Extensin gene region (9:232692750-232694483_ID: PHT73052.1). Highlighted in yellow the mutation site (Insertion of adenine T -> TA in genotypes from Campania). Flanking primers are highlighted in red (ExtF) and blue (reverse complement of ExtR) font, respectively.
