# Supplementary material for: Whole-genome resequencing reveals genomic footprints of Italian sweet and hot pepper heirlooms giving insight into genes underlying key agronomic and qualitative traits
Source: BMC Genom Data. 2022 Mar 25;23:21. doi: 10.1186/s12863-022-01039-9 (PMC8957157; doi:10.1186/s12863-022-01039-9)
Supplement: Supplementary file 8 — Additional file 8: Table S1. Mean coverage of each chromosome in all genomes analyzed. [file 12863_2022_1039_MOESM8_ESM.docx]

| **Chr** | **Reference chr length (bp)** | **CDT** | **PAP** | **CIL** | **SIG** |
| --- | --- | --- | --- | --- | --- |
| 1 | 309102287 | 20.83 | 19.63 | 21.74 | 26.56 |
| 2 | 169555599 | 19.85 | 19.28 | 21.70 | 25.96 |
| 3 | 282780301 | 20.19 | 18.86 | 21.70 | 25.97 |
| 4 | 240120734 | 20.64 | 19.65 | 22.30 | 26.68 |
| 5 | 238597879 | 20.04 | 19.29 | 21.84 | 25.99 |
| 6 | 242241289 | 20.93 | 19.61 | 22.24 | 26.47 |
| 7 | 251293532 | 20.45 | 19.32 | 21.95 | 26.23 |
| 8 | 142366738 | 21.72 | 19.97 | 23.28 | 27.28 |
| 9 | 271082670 | 19.47 | 17.94 | 21.22 | 25.74 |
| 10 | 233321800 | 22.33 | 20.82 | 23.69 | 27.56 |
| 11 | 266870110 | 20.36 | 19.04 | 22.05 | 26.10 |
| 12 | 250929874 | 20.25 | 19.27 | 21.71 | 25.90 |

**Table S1**. Mean coverage of each chromosome in all genomes analyzed.
