# Supplementary material for: Whole-genome resequencing reveals genomic footprints of Italian sweet and hot pepper heirlooms giving insight into genes underlying key agronomic and qualitative traits
Source: BMC Genom Data. 2022 Mar 25;23:21. doi: 10.1186/s12863-022-01039-9 (PMC8957157; doi:10.1186/s12863-022-01039-9)
Supplement: Supplementary file 9 — Additional file 9: Table S2. Number of small (SNPs and INDELS) and big variants (Deletions, Duplications and Inversions). The number of new contigs is also shown. [file 12863_2022_1039_MOESM9_ESM.docx]

| **Features** | **CDT** | **PAP** | **CIL** | **SIG** |
| --- | --- | --- | --- | --- |
| SNP | 7,664,524 | 7,373,882 | 5,987,242 | 8,233,170 |
| Small indels | 475,286 | 471,421 | 397,056 | 535,349 |
| Deletions | 3,891 | 3,850 | 3,411 | 4,455 |
| Inversions | 40 | 45 | 33 | 36 |
| Duplications | 50 | 45 | 52 | 78 |
| New contigs | 35 | 46 | 53 | 62 |

**Table S2.** Number of small (SNPs and INDELS) and big variants (Deletions, Duplications and Inversions). The number of new contigs is also shown.
