# Supplementary material for: Whole-genome resequencing reveals genomic footprints of Italian sweet and hot pepper heirlooms giving insight into genes underlying key agronomic and qualitative traits
Source: BMC Genom Data. 2022 Mar 25;23:21. doi: 10.1186/s12863-022-01039-9 (PMC8957157; doi:10.1186/s12863-022-01039-9)
Supplement: Supplementary file 10 — Additional file 10: Table S3. Number of inversions per chromosome identified in the four genomes investigated. [file 12863_2022_1039_MOESM10_ESM.docx]

| **chr** | **CDT** | **PAP** | **CIL** | **SIG** |
| --- | --- | --- | --- | --- |
| 1 | 2 | 4 | 3 | 3 |
| 2 | 2 | 3 | - | 2 |
| 3 | 5 | 6 | 7 | 4 |
| 4 | 2 | 2 | 2 | 3 |
| 5 | 5 | 5 | 2 | 3 |
| 6 | 1 | 2 | 1 | 1 |
| 7 | 6 | 5 | 5 | 4 |
| 8 | 1 | 1 | 2 | 1 |
| 9 | 6 | 7 | 6 | 3 |
| 10 | - | 1 | 1 | 4 |
| 11 | 9 | 8 | 4 | 6 |
| 12 | 1 | 1 | - | 2 |

**Table S3.** Number of inversions per chromosome identified in the four genomes investigated.
