# Supplementary material for: Whole-genome resequencing reveals genomic footprints of Italian sweet and hot pepper heirlooms giving insight into genes underlying key agronomic and qualitative traits
Source: BMC Genom Data. 2022 Mar 25;23:21. doi: 10.1186/s12863-022-01039-9 (PMC8957157; doi:10.1186/s12863-022-01039-9)
Supplement: Supplementary file 11 — Additional file 11: Table S4. Number of deletions per chromosome identified in the four genomes investigated. [file 12863_2022_1039_MOESM11_ESM.docx]

| **chr** | **CDT** | **PAP** | **CIL** | **SIG** |
| --- | --- | --- | --- | --- |
| 1 | 445 | 448 | 457 | 528 |
| 2 | 217 | 208 | 213 | 262 |
| 3 | 301 | 357 | 258 | 302 |
| 4 | 212 | 218 | 220 | 248 |
| 5 | 308 | 309 | 278 | 364 |
| 6 | 236 | 264 | 294 | 322 |
| 7 | 365 | 338 | 359 | 393 |
| 8 | 112 | 104 | 113 | 119 |
| 9 | 522 | 516 | 404 | 446 |
| 10 | 272 | 225 | 247 | 579 |
| 11 | 573 | 563 | 263 | 512 |
| 12 | 225 | 207 | 208 | 257 |

**Table S4.** Number of deletions per chromosome identified in the four genomes investigated.
