# Supplementary material for: Whole-genome resequencing reveals genomic footprints of Italian sweet and hot pepper heirlooms giving insight into genes underlying key agronomic and qualitative traits
Source: BMC Genom Data. 2022 Mar 25;23:21. doi: 10.1186/s12863-022-01039-9 (PMC8957157; doi:10.1186/s12863-022-01039-9)
Supplement: Supplementary file 12 — Additional file 12: Table S5. Number of duplications per chromosome identified in the four genomes investigated. [file 12863_2022_1039_MOESM12_ESM.docx]

| **chr** | **CDT** | **PAP** | **CIL** | **SIG** |
| --- | --- | --- | --- | --- |
| 1 | 6 | 4 | 7 | 10 |
| 2 | 5 | 1 | 4 | 4 |
| 3 | 6 | 7 | 3 | 10 |
| 4 | 3 | 3 | 4 | 6 |
| 5 | 4 | 2 | 1 | 7 |
| 6 | 1 | 2 | 6 | 6 |
| 7 | 5 | 3 | 4 | 6 |
| 8 | - | - | 1 | - |
| 9 | 3 | 6 | 4 | 5 |
| 10 | 6 | 6 | 7 | 8 |
| 11 | 5 | 6 | 6 | 8 |
| 12 | 2 | 4 | 4 | 5 |

**Table S5.** Number of duplications per chromosome identified in the four genomes investigated.
