# Supplementary material for: Whole-genome resequencing reveals genomic footprints of Italian sweet and hot pepper heirlooms giving insight into genes underlying key agronomic and qualitative traits
Source: BMC Genom Data. 2022 Mar 25;23:21. doi: 10.1186/s12863-022-01039-9 (PMC8957157; doi:10.1186/s12863-022-01039-9)
Supplement: Supplementary file 13 — Additional file 13: Table S6. BUSCO statistics for the four pepper genomes using Eukaryote, Solanales and Viridiplantae databases. [file 12863_2022_1039_MOESM13_ESM.docx]

**Table S6.** BUSCO statistics for the four pepper genomes using *Eukaryote, Solanales* and *Viridiplantae* databases*.*

| **Database** | **Features** | **CDT** | **PAP** | **CIL** | **SIG** |
| --- | --- | --- | --- | --- | --- |
| *Eukaryote* db | Complete BUSCOs (C) | 93.7% | 92.9% | 96.9% | 94.5% |
|  | Complete and single-copy BUSCOs (S) | 74.1% | 74.5% | 80.8% | 80.0% |
|  | Complete and duplicated BUSCOs (D) | 19.1% | 18.4% | 16.1% | 14.5% |
|  | Fragmented BUSCOs (F) | 4.7% | 2.7% | 1.2% | 2.7% |
|  | Missing BUSCOs (M) | 1.6% | 4.4% | 1.9% | 2.8% |
| *Solanales* db | Complete BUSCOs (C) | 91.4% | 87.6% | 91.4% | 89.1% |
|  | Complete and single-copy BUSCOs (S) | 81.3% | 78.4% | 83.8% | 81.2% |
|  | Complete and duplicated BUSCOs (D) | 10.1% | 9.2% | 7.6% | 7.9% |
|  | Fragmented BUSCOs (F) | 2.4% | 2.6% | 2.3% | 3.1% |
|  | Missing BUSCOs (M) | 6.2% | 9.8% | 6.3% | 7.8% |
| *Viridiplantae* db | Complete BUSCOs (C) | 94.6% | 91.3% | 94.6% | 90.6% |
|  | Complete and single-copy BUSCOs (S) | 80.0% | 80.2% | 83.8% | 78.6% |
|  | Complete and duplicated BUSCOs (D) | 13.9% | 11.1% | 10.8% | 12.0% |
|  | Fragmented BUSCOs (F) | 3.8% | 4.7% | 3.5% | 6.6% |
|  | Missing BUSCOs (M) | 1.6% | 4.0% | 1.9% | 2.8% |
