# Supplementary material for: Whole-genome resequencing reveals genomic footprints of Italian sweet and hot pepper heirlooms giving insight into genes underlying key agronomic and qualitative traits
Source: BMC Genom Data. 2022 Mar 25;23:21. doi: 10.1186/s12863-022-01039-9 (PMC8957157; doi:10.1186/s12863-022-01039-9)
Supplement: Supplementary file 14 — Additional file 14: Table S7. Statistics of SNPs/indels effects within each of the four genotypes. All variants (heterozygous and homozygous) are reported. [file 12863_2022_1039_MOESM14_ESM.docx]

**Table S7.** Statistics of SNPs/indels effects within each of the four genotypes. All variants (heterozygous and homozygous) are reported.

| **SNPeff impact** | **CDT** | **PAP** | **CIL** | **SIG** |
| --- | --- | --- | --- | --- |
| High | 2.956 | 2.755 | 2.408 | 2.776 |
| Low | 17.067 | 15.640 | 14.637 | 16.273 |
| Moderate | 28.321 | 26.068 | 23.657 | 27.029 |
| Modifier | 8,400.671 | 8,050.734 | 6,643.590 | 8,892.438 |
| Total SNP | 8,449.015 | 8,095.197 | 6,684.292 | 8,938.516 |
