# Supplementary material for: Whole-genome resequencing reveals genomic footprints of Italian sweet and hot pepper heirlooms giving insight into genes underlying key agronomic and qualitative traits
Source: BMC Genom Data. 2022 Mar 25;23:21. doi: 10.1186/s12863-022-01039-9 (PMC8957157; doi:10.1186/s12863-022-01039-9)
Supplement: Supplementary file 15 — Additional file 15: Table S8. Number of genes showing private SNPs/Indels in genomes from Campania and Calabria, respectively. [file 12863_2022_1039_MOESM15_ESM.docx]

| **Chr** | **N° genes Campania** | **N° genes Calabria** |
| --- | --- | --- |
| 1 | 89 | 1059 |
| 2 | 218 | 1,700 |
| 3 | 103 | 2,896 |
| 4 | 12 | 364 |
| 5 | 20 | 95 |
| 6 | 239 | 321 |
| 7 | 42 | 547 |
| 8 | 12 | 590 |
| 9 | 1,535 | 951 |
| 10 | 46 | 257 |
| 11 | 57 | 805 |
| 12 | 28 | 471 |
| Total | 2,402 | 10,057 |

**Table S8.** Number of genes showing private SNPs/Indels in genomes from Campania and Calabria, respectively.
