# Supplementary material for: Whole-genome resequencing reveals genomic footprints of Italian sweet and hot pepper heirlooms giving insight into genes underlying key agronomic and qualitative traits
Source: BMC Genom Data. 2022 Mar 25;23:21. doi: 10.1186/s12863-022-01039-9 (PMC8957157; doi:10.1186/s12863-022-01039-9)
Supplement: Supplementary file 16 — Additional file 16: Table S9. SNPeff statistics regarding private SNPs in genomes from Campania and Calabria, respectively. [file 12863_2022_1039_MOESM16_ESM.docx]

**Table S9.** SNPeff statistics regarding private SNPs in genomes from Campania and Calabria, respectively.

| **Effect** | **No. SNPs**  **Campania** | **No. SNPs Calabria** |
| --- | --- | --- |
| Low | 30 | 175 |
| Moderate | 67 | 285 |
| High | 10 | 32 |
| Modifier | 2,294 | 9,564 |
| Total | 2,402 | 10,057 |
